# *In Situ* Design of a Nanostructured Interface between NiMo and CuO Derived from Metal–Organic Framework for Enhanced Hydrogen Evolution in Alkaline Solutions

**DOI:** 10.1021/acsami.3c17588

**Published:** 2024-02-20

**Authors:** Ebrahim Sadeghi, Sanaz Chamani, Ipek Deniz Yildirim, Emre Erdem, Naeimeh Sadat Peighambardoust, Umut Aydemir

**Affiliations:** † Koç University Boron and Advanced Materials Applications and Research Center (KUBAM), Sariyer, Istanbul 34450, Turkey; ‡ Graduate School of Sciences and Engineering, Koç University, Sariyer, Istanbul 34450, Turkey; § Faculty of Engineering and Natural Sciences, Materials Science and Nano Engineering, Sabanci University, Istanbul 34956, Turkey; ∥ Sabanci University Integrated Manufacturing Technologies Research and Application Center, Composite Technologies Center of Excellence, Teknopark Istanbul, Pendik, Istanbul 34906, Turkey; ⊥ Department of Chemistry, Koç University, Sariyer, Istanbul 34450, Turkey

**Keywords:** electrocatalysis, hydrogen evolution reaction, metal−organic framework, transition metal oxides, nanostructured interface

## Abstract

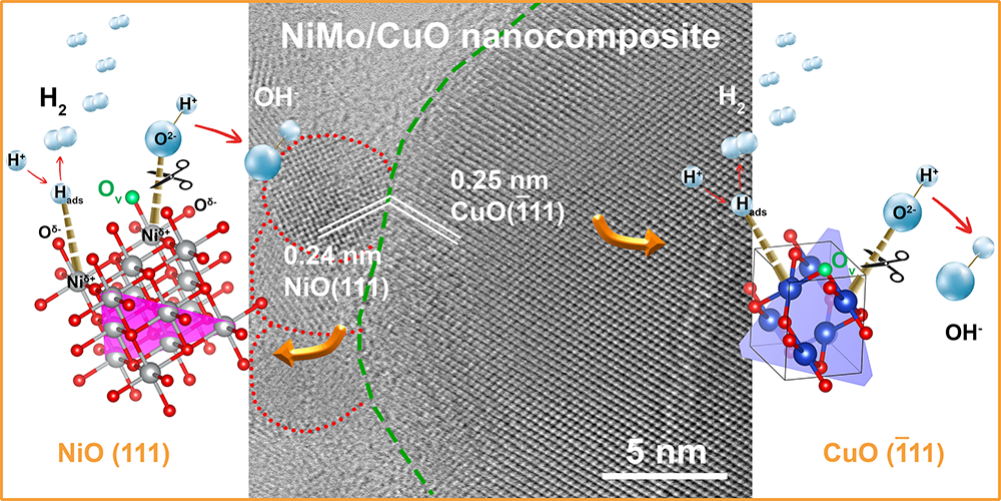

Hydrogen shows great promise as a carbon-neutral energy carrier that can significantly mitigate global energy challenges, offering a sustainable solution. Exploring catalysts that are highly efficient, cost-effective, and stable for the hydrogen evolution reaction (HER) holds crucial importance. For this, metal–organic framework (MOF) materials have demonstrated extensive applicability as either a heterogeneous catalyst or catalyst precursor. Herein, a nanostructured interface between NiMo/CuO@C derived from Cu-MOF was designed and developed on nickel foam (NF) as a competent HER electrocatalyst in alkaline media. The catalyst exhibited a low overpotential of 85 mV at 10 mA cm^–2^ that rivals that of Pt/C (83 mV @ 10 mA cm^–2^). Moreover, the catalyst’s durability was measured through chronopotentiometry at a constant current density of −30, −100, and −200 mA cm^–2^ for 50 h each in 1.0 M KOH. Such enhanced electrocatalytic performance could be ascribed to the presence of highly conductive C and Cu species, the facilitated electron transfer between the components because of the nanostructured interface, and abundant active sites as a result of multiple oxidation states. The existence of an ionized oxygen vacancy (O_v_) signal was confirmed in all heat-treated samples through electron paramagnetic resonance (EPR) analysis. This revelation sheds light on the entrapment of electrons in various environments, primarily associated with the underlying defect structures, particularly vacancies. These trapped electrons play a crucial role in augmenting electron conductivity, thereby contributing to an elevated HER performance.

## 1 Introduction

Molecular hydrogen (H_2_) is widely recognized as a highly promising fuel option for driving the development of a sustainable “green” economy in the future.^1,2^ Electrochemical water splitting (EWS), a powerful method for producing high-purity hydrogen, represents a highly effective and sophisticated energy conversion technology.^3^ Its remarkable potential extends to alleviating the pressing global energy conundrum and addressing the urgent environmental crisis, thus presenting an elegant pathway toward sustainable solutions.^4^ Despite its promise, the overall energy efficiency of EWS faces a significant setback due to the sluggish kinetics of the hydrogen evolution reaction (HER) under alkane conditions. Thus, it is vital to create catalysts that excel in performance while being highly efficient for a sustainable future.^5,6^ Over the past decade, a wave of exploration has unfolded in the realm of electrocatalysis, wherein first-row transition metals like Fe, Co, and Ni have taken center stage as excellent candidates for the EWS. Regrettably, the vibrant domain of Cu-based catalysts, endowed with their earth-abundant nature, has remained relatively unexplored when it comes to unraveling their intricate electrocatalytic properties.^1^

Cu-based compounds show great promise for electrocatalytic applications, thanks to their abundance and cost-effectiveness.^7,8^ The redox-active properties of copper are well-established, demonstrated by its ability to undergo oxidation from Cu(II) to Cu(III) and reduction from Cu(II) to Cu(I) and Cu(0).^9^ These well-established redox properties of copper oxide further underline its significance in various contexts.^7,10^ Despite their potential, Cu-based oxide electrocatalysts still exhibit unsatisfactory performance in the HER due to their simplistic configuration and morphological characteristics. These factors contribute to the agglomeration of active substances and severely limit the availability of sparsely exposed active sites for catalytic processes.^11^ Hence, to enhance the benefits offered by Cu-based catalysts, designing and synthesizing porous materials with copper as a key component are both meaningful and desirable. These materials should possess high specific surface areas and a significant proportion of exposed active sites, allowing for the development of highly efficient electrocatalysts through facile and controllable synthesis routes.^1^

Organic–inorganic hybrid materials including metal–organic frameworks (MOFs) and zeolitic imidazolate frameworks (ZIFs) offer promising features due to their adjustable porosity and structure. Among these, MOF-derived materials, characterized by a controllable surface area and functional groups, are highly potential for electrocatalytic applications.^12,13^ Notably, Cu-BDC MOF (CCDC#687690), a well-known MOF, is synthesized from copper nitrate and the widely available, environmentally safe terephthalic acid (BDC).^14,15^ Cu-BDC demonstrates compelling traits, such as strong stability in aqueous environments within diverse pH ranges, attributed to the robust coordination interaction between copper cations and the −COOH group of BDC.^14^ In some reports, however, it is claimed that the direct usage of MOFs as catalysts is not desirable due to their poor conductivity, limited accessibility of active metallic sites, and low stability in aqueous environments.^16,17^ Consequently, numerous endeavors have been undertaken to promote the electrical conductivity of materials based on MOFs through methods such as heat treatment at high temperature^15,18^ or combining them with conductive supports.^17,19^

According to the literature, the MOF-derived transition metal oxide (TMO) catalysts tend to undergo self-aggregation, resulting in the loss of exposed active sites and a concomitant reduction in mass transfer during electrocatalysis.^20,21^ Hence, there is keen anticipation and a challenging yet intriguing task ahead—exploring alternative approaches or carefully selecting suitable MOF precursors. This is paramount for producing novel electrocatalysts based on TMOs with the promise of significantly improved performance for the HER.^20^ In recent reports, it has been revealed that enhancing the performance of Cu-based materials for the HER is achievable through the meticulous design of their compositions, structures, and morphologies.^22,23^ One of these strategies that stands out among others could be the heat treatment of Cu-MOF with which the carbon-decorated copper oxide is attainable with a flexible structure and tunable porosity at molecular level.^24,25^ Carbon-decorated TMOs, derived from MOFs, emerge as promising candidates for the HER, attributed to their enhanced electrocatalytic stability even under harsh reaction conditions.^26,27^

In the pursuit of high-performance electrode materials, in addition to the above-mentioned approach, significant endeavors have been dedicated to the strategic design of electrode materials, featuring precisely defined micro/nanostructures through the fabrication of composites and hybrid materials.^28^ Recently, we reported a heterostructured HER electrocatalyst consisting of oxygen vacancy-confined CoMoO_4_ and NiMo alloy that showcased its high activity and durability under alkaline electrolytes.^29^ It is believed that incorporating Ni with other metals allows for the fine-tuning of the electronic structure, consequently enhancing catalytic activity, examples being NiFe, NiCo, and NiMo alloys.^30−32^ Particularly, NiMo alloy is well-established as a highly effective electrocatalyst for the HER in alkaline electrolytes.^30,33^ Hence, through the deliberate design of heterointerfaces, it becomes feasible to effectively adjust electron distribution, generate numerous active sites, and amplify the chemical adsorption capability of the catalyst. This, in turn, leads to a significant enhancement in electrochemical activity. Additionally, elevating the number of exposed active sites can further augment the electrocatalytic activity.^6,7^ However, the exploration and documentation of hybrid systems derived from Cu-MOFs have been scarce and limited.

In this study, we introduce a novel electrocatalyst, Cu-BDC MOF-derived NiMo/CuO, for alkaline HER. The fabrication process involved a hydrothermal procedure to grow the materials directly on a Cu-coated nickel foam (NF) substrate, followed by a heat treatment step to produce the final composite on the NF skeleton. For the first time, we introduce an *in situ* grown interface formed between NiMo and CuO through heat treatment. The resulting hybrid material enhances charge transfer within the structure, further amplified by the presence of oxygen vacancies in both components. The distinctive interface between the two components significantly enhanced electron transfer during the reduction process. As a result, the catalyst exhibited exceptional performance, attaining a current density of 10 mA cm^–2^ with a minimal overpotential of 85 mV. Additionally, it sustained high current densities of −30, −100, and −200 mA cm^–2^ individually, each for 50 h.

## 2 Experimental Section

### 2.1 Catalyst Preparation

#### 2.1.1 Materials

1,4-Benzenedicarboxylic acid [terephthalic acid/1,4-BDC, Sigma-Aldrich, 98%], *N*,*N-*dimethylformamide [DMF, Sigma-Aldrich], polyvinylpyrrolidone (PVP, Sigma-Aldrich), nickel chloride hexahydrate (NiCl_2_·6H_2_O, Sigma-Aldrich, 99.9% trace metals basis), sodium molybdate dihydrate (Na_2_MoO_4_·2H_2_O, Sigma-Aldrich, ACS reagent, ≥99%), sodium borohydride (NaBH_4_, Sigma-Aldrich), copper(II) sulfate pentahydrate (CuSO_4_·5H_2_O, ACS reagent, ≥98.0%), and copper(II) nitrate trihydrate (Cu(NO_3_)_2_·3H_2_O, Sigma-Aldrich, puriss. p.a., 99–104%) were purchased and used without any prior treatment.

#### 2.1.2 Synthesis of NiMo Nanoparticles

We discussed the preparation protocol for NiMo nanoparticles (NPs) in our recent publication.^29^ Briefly, the synthesis of NiMo NPs involved dissolving 1g of PVP in 50 mL of deionized (DI) water, adding Ni and Mo precursors (NiCl_2_·6H_2_O and Na_2_MoO_4_·2H_2_O), followed by the addition of NaBH_4_. After rigorous stirring, the resulting black suspension was washed, dried, and heated under an air atmosphere at 500 °C for 2 h to obtain crystalline NiMo NPs.

#### 2.1.3 Synthesis of NiMo/Cu-BDC MOF and NiMo/CuO Derived from Cu-BDC MOF on NF

The commercial NF (1.5 cm × 3 cm) underwent a thorough treatment process to eliminate surface contaminants. This included ultrasonication with a 2.0 M HCl solution, followed by exposure to acetone, DI water, and ethanol, each for 10 min. The NF was then briefly dried in open air to ensure the removal of oxides and other impurities. The treated NF pieces were collected and stored in the glovebox (MBraun Labmaster Pro DP glovebox, O_2_ and H_2_O level <0.1 ppm) to serve as substrates for catalyst growth. To grow Cu-BDC on the NF, the first step involved electrodeposition of Cu on the NF using a 0.05 M CuSO_4_·5H_2_O solution. A three-electrode cell configuration was employed, with a piece of Cu foil as the counter electrode and the Calomel electrode as the reference electrode. The pretreated NF, which was previously subjected to heat treatment under an ambient environment at 550 °C for 2 h, utilized as the working electrode. The electrodeposition process was carried out via a chronopotentiometry experiment with a current of −0.02 A applied for 2.5 h. The optical microscope image of Cu-coated NF under polarized light can be seen in Figure S1.

**Scheme 1:**
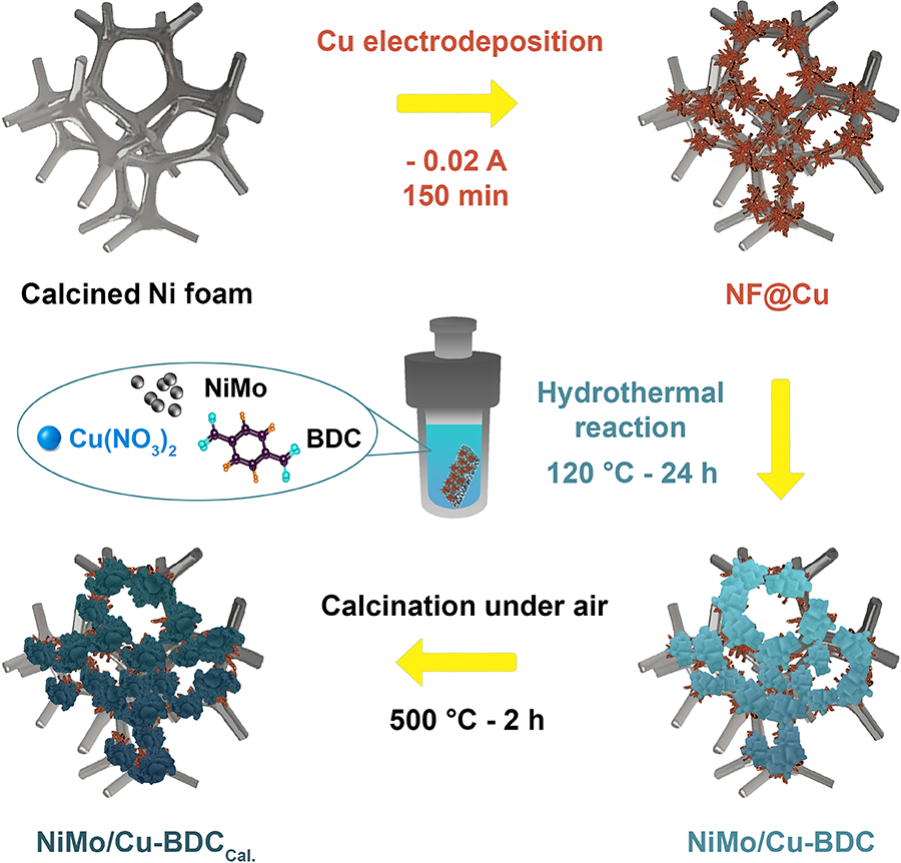
Schematic Illustration of the Preparation Procedure of NiMo/Cu-BDC_Cal._ (CuO Derived from Cu-BDC MOF) on NF

For the fabrication of NiMo/Cu-BDC on the Cu-deposited NF, a solution was prepared by dissolving 2 mmol Cu(NO_3_)_2_·3H_2_O and 2 mmol terephthalic acid separately in 20 mL of DMF. The two solutions were mixed and stirred for 30 min, resulting in a blue solution. Subsequently, a quantified amount of NiMo (10 wt %) was added to the mixture and stirred for another 30 min, creating a black solution. To ensure thorough dissolution and mixing, the black solution was ultrasonicated for an additional 30 min. The prepared mixture was then poured into a 50 mL Teflon-lined stainless-steel autoclave, where the previously Cu-deposited NF was added. The autoclave was tightly sealed and placed in an oven, maintained at 120 °C for 24 h. After cooling down to room temperature naturally, the product was rinsed with DI water and ethanol before being vacuum-dried overnight at 80 °C. The final product was annealed at 500 °C for 2 h in air. The schematic illustration in Scheme 1 briefly describes the synthesis process. The pristine Cu-BDC was prepared following the identical steps without the addition of NiMo to the solution. In this article, for ease of reference, we assign the labels NiMo_Cal._, Cu-BDC_Cal._, and NiMo/Cu-BDC_Cal._ to the NiMo, Cu-BDC, and NiMo/Cu-BDC samples after undergoing heat treatment, respectively.

#### 2.1.4 Preparation of Pt/C on NF

One mg of commercial 20 wt % Pt/C was ultrasonically dispersed in a 300 μL ethanol and 200 DI water solution for 30 min. Subsequently, 10 μL of Nafion solution was added and sonicated for an additional 30 min to create a homogeneous ink. This ink was then pipetted onto an NF substrate, achieving a mass loading of about 1 mg cm^–2^, to be comparable with *in situ* fabricated catalysts.

### 2.2 Structural Characteristics

The crystal phase and purity of the synthesized materials were assessed using X-ray diffraction (XRD) analysis performed with a Rigaku Mini Flex 600 instrument equipped with Cu Kα radiation (λ = 1.5418 Å). The morphology was characterized via field emission-scanning electron microscopy (FE-SEM, Zeiss Ultra Plus), coupled with an energy-dispersive X-ray spectroscopy detector (EDS, Bruker Xflash 5010) offering a spectral resolution of 123 eV. For a comprehensive microstructural analysis, including selected area electron diffraction (SAED), examination of lattice fringes, high-angle annular dark field-scanning transmission electron microscopy (HAADF-STEM) images, and corresponding EDS-STEM mappings, high-resolution transmission electron microscopy (HR-TEM; Hitachi HF5000 200 kV (S)TEM) was employed. The surface composition and oxidation states were probed using X-ray photoelectron spectroscopy (XPS, Thermo Scientific K-Alpha) with an Al Kα monochromator source emitting at 1486.6 eV. The XPS spectra were appropriately calibrated with respect to the binding energy (BE) of C 1s, set at 284.50 eV. The vibrational modes of the molecules were investigated through Raman spectroscopy employing a Raman microscope (RENISHAW INVIA) with a 532 nm excitation laser source. X-Band (9.65 GHz) electron paramagnetic resonance (EPR) measurements were carried out at room temperature via a Bruker EMX Nano benchtop spectrometer with 2 G modulation amplitude and 0.3162 mW microwave power for 50 scans. Identification of functional groups was accomplished using Fourier transform-infrared spectroscopy (FT-IR, JASCO 6800 full vacuum and FT-IR microscope). The Brunauer–Emmett–Teller (BET) specific surface area was ascertained through N_2_ adsorption–desorption isotherms, utilizing a Micromeritics ASAP 2010 instrument.

### 2.3 Electrochemical Characteristics

All electrochemical investigations were executed using an AutoLab Potentiostat Galvanostat (PGSTAT302N, fabricated in The Netherlands) within a conventional three-electrode setup immersed in an alkaline medium (1.0 M KOH). The reference electrode employed was a reversible hydrogen electrode (RHE, HydroFlex), while a platinum (Pt) spring was implemented as the counter electrode. Working electrodes were composed of catalysts grown on nanostructured substrates (0.5 cm × 1 cm). Linear sweep voltammetry (LSV) profiles were recorded within the region of HER potentials (ranging from 0 to −1 V) at a scanning rate of 5 mV s^–1^. HER overpotentials were evaluated at current densities of 10 and 50 mA cm^–2^. The acquired LSV data were subsequently transformed into Tafel plots. Electrochemical double layer capacitance (*C*_dl_), pertinent to cyclic voltammetry (CV) conducted at varying scan rates (0.02, 0.04, 0.06, 0.08, 0.1, 0.12, 0.14, 0.16, 0.18, and 0.2 V s^–1^) across the potential window from 0.7 to 0.8 V vs RHE, was used to determine the electrochemically active surface area (ECSA). To assess long-term HER stability, chronopotentiometry tests were conducted at a constant applied current density of −30, −100, and −200 mA cm^–2^, each maintained for a duration of 50 h. Electrochemical impedance spectroscopy (EIS) measurements were conducted at −300 mV vs RHE, spanning a frequency spectrum from 100 kHz to 0.1 Hz. All electrochemical data were presented without considering *iR* correction, as the calculated *iR* drop was determined to be 11.7 Ω, which exhibited negligible influence on the outcomes.

## 3 Results and Discussion

### 3.1 Structural Studies

**Figure 1:**
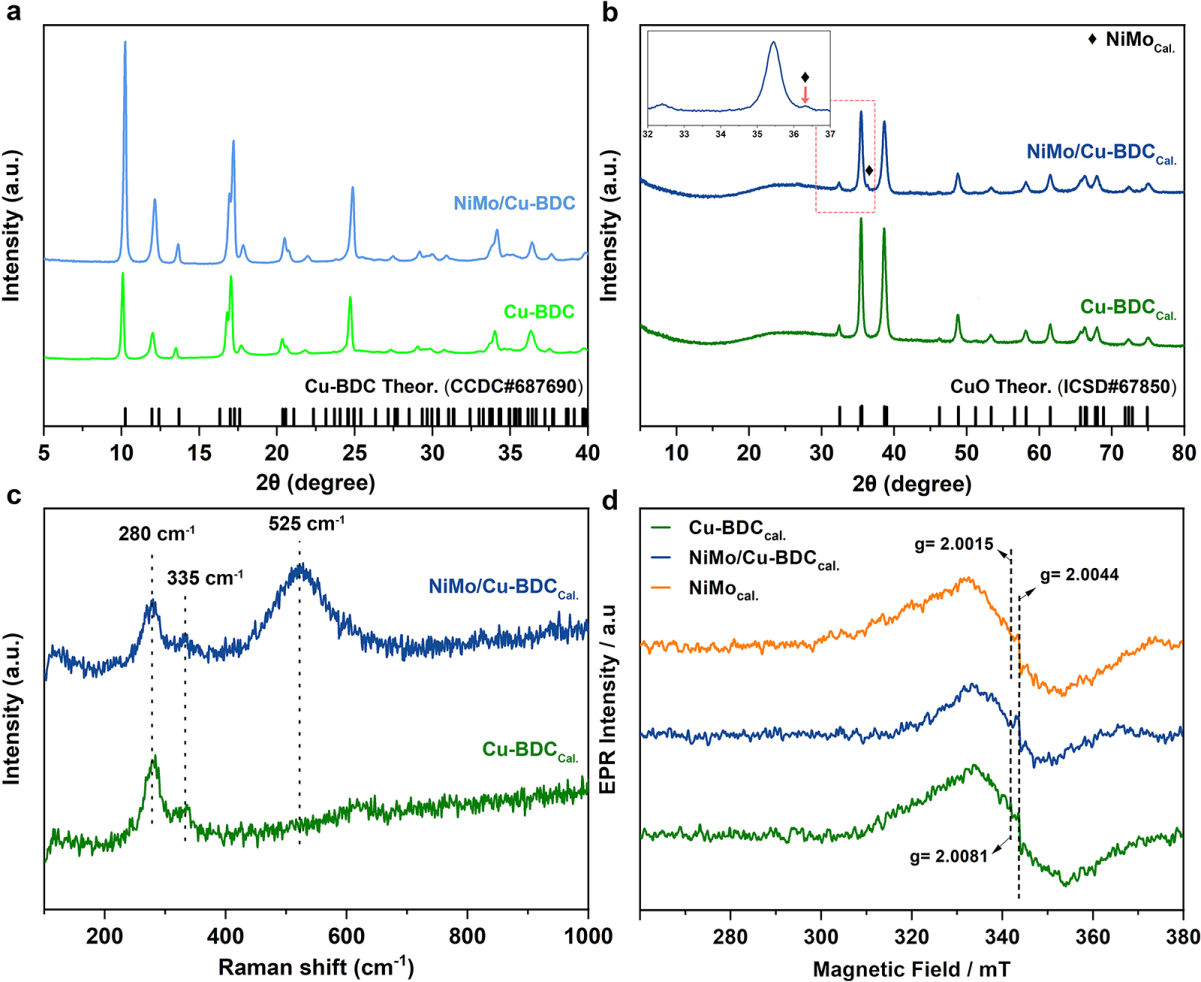
Structural and spectroscopic characterizations. (a) XRD patterns of Cu-BDC and NiMo/Cu-BDC stripped off from NF, (b) XRD patterns of Cu-BDC_Cal._ and NiMo/Cu-BDC_Cal._ stripped off from NF, (c) Raman spectra for Cu-BDC_Cal._ and NiMo/Cu-BDC_Cal._, and (d) X-band EPR spectra for calcined samples.

The XRD technique was employed to investigate the crystalline composition of the as-obtained samples. Figure S2 illustrates the XRD patterns of NiMo NPs before and after heat treatment. The single broad peak at 2θ around 45° for NiMo before heat treatment is indicative of an amorphous structure. On the other hand, the XRD pattern of NiMo after heat treatment shows a distinct difference. Our analysis confirmed the transformation of NiMo to NiO (ICSD#9866) and MoO_2_ (at 2θ = 26° and 37.2° marked by black heart solid) after applying heat treatment. The XRD results of Cu-BDC and NiMo/Cu-BDC scratched off from the substrate are provided in Figure 1a. It can be found that all the reflections are attributed to the simulated pattern from the single crystal data of the Cu-BDC (CCDC#687690). Finally, the diffraction peaks of Cu-BDC_Cal._ and NiMo/Cu-BDC_Cal._ can be visualized in Figure 1b. According to these results, both MOF-bearing samples transform to CuO (ICSD#67850) once the heat treatment is applied. There are no other forms of copper oxide in these samples, demonstrating the high purity of the final products. The single minor peak (marked by tilted square solid) appeared within the diffraction pattern of NiMo/Cu-BDC_Cal._, displaying the presence of NiO in the sample. It is noteworthy that the weak and broad peak at 2θ = 26° for both samples after calcination confirms the carbonization as a result of the heat treatment, corresponding to the (002) plane of the graphitized carbon.

Raman spectroscopy was used to monitor structural evolution during the thermal treatment process of NiMo, Cu-BDC, and NiMo/Cu-BDC. The Raman characteristic peaks for both Cu-BDC and NiMo/Cu-BDC are displayed in Figure S3a. The architecture of Cu-BDC exhibits a three-dimensional (3D) network, interconnecting the two-dimensional (2D) layers comprising porous copper(II) dicarboxylate—so-called copper paddle wheels—by the DMF solvent (refer to Figure S4). In the higher frequency range (≥900 cm^–1^), the Raman bands predominantly arise from the vibrational modes of the BDC linkers. Specifically, the band observed at 1620.7 cm^–1^ corresponds to the phenyl mode (C=C stretching mode of BDC). Additionally, the feature at 1530.9 cm^–1^ can be attributed to the asymmetric in-plane vibration of COO, while the bands at 1436.3 and 1419 cm^–1^ are indicative of the symmetric vibration of COO. In the low-frequency region (100–750 cm^–1^), Cu-BDC and NiMo/Cu-BDC before heat treatment exhibited more complex Raman spectra because of the presence of vibrational modes of metal (Cu) oxide clusters. Three bands at 456.8, 400, and 326 cm^–1^ are attributed to Cu–O species.^34^ As shown in Figure S3b, a set of strong Raman peaks was observed for NiMo after heat treatment. The bands observed at 939.1, 893.7, and 820.3 cm^–1^ are ascribed to the Mo–O vibrations and are in good agreement with previous reports.^35^ The bands positioned at 367 and 341.4 cm^–1^ correspond to symmetric torsional vibrations of O–Mo–O.^29^ Interestingly, the new weak peak appeared at 119.6 cm^–1^ and could be attributed to the lattice deformation mode for MoO_2_ species.^35^ Finally, the Raman spectroscopy for Cu-BDC_Cal._ and NiM/Cu-BDC_Cal._ demonstrated three signals at 280, 335, and 525 cm^–1^ (shown in Figure 1c) corresponding to the A_g_, B_g_^(^^1)^, and B_g_^(^^2)^ modes of the CuO single crystal, respectively.^36^

The utilization of EPR analysis proves highly effective in discerning intricate defect structures within the samples under study. The observed EPR spectra in Figure 1d consistently exhibit a broad Gaussian line, primarily attributed to dipolar interactions (spin–spin interactions), notably arising from Cu–Cu interactions (g = 2.0015 and 2.0081) prevalent within the heat-treated materials, consistent with the g-factor for CuO reported in the literature.^37^ Despite this dominant interaction, the presence of an ionized oxygen vacancy (O_v_) signal across all samples is evident, elucidating the entrapment of electrons in diverse environments attributable to the underlying defect structures, predominantly vacancies.

The emergence of a distinct, sharp, and minutely varied signal, characterized by slightly different g-factors (g = 2.0015, 2.0044, and 2.0081) in each sample, signifies the nuanced variations in the trapping of electrons within the distinct defect environments, underscoring the sensitivity of EPR in delineating the intricate landscape of defect-induced phenomena within these materials. The g-factors are very close to the typical free electron g-value, which is 2.0023. When the g-factor of a defect center closely resembles that of a free electron, it implies a limited influence of spin–orbit coupling within the defect’s electronic structure. Typically, spin–orbit coupling leads to alterations in the g-factor, shifting it from the free electron value due to the interaction between the electron’s spin and its orbital angular momentum. The similarity in g-factors suggests that, within this particular defect center, the spin–orbit interaction might be relatively weak or negligible. This could indicate that the defect’s electronic configuration or the specific symmetry of the defect site minimally affects the electron’s spin–orbit coupling compared to the influence of other factors, such as its spin–spin interactions or local magnetic environment.

According to previous studies, the successful oxygen vacancy engineering of metal oxides is responsible for their significantly enhanced electronic conductivity.^29,38^ In this regard, oxygen vacancy modification introduces some new electronic states energy near the Fermi level, which directly leads to higher electronic conductivity of metal oxides. Notably, it is well-accepted that the inherently poor electronic conductivity of metal oxides is one of the key problems that hamper their activity and durability. This issue can be overcome by introducing oxygen defects, and in turn, the trapped electrons greatly contribute to the HER activity and durability of metal oxides.^38^ Based on XRD results, NiMo NPs, following heat treatment, predominantly transformed into the NiO phase, accompanied by a small amount of MoO_2_. This transformation suggests that, upon removal of Mo from the amorphous structure of NiMo, it reacts with and binds to oxygen. Consequently, the resulting NiO exhibits oxygen deficiency. Therefore, the final product can also be termed NiO_1–*x*_, where *x* is not easily determined.

Figure S5a represents the FT-IR spectra of prepared Cu-BDC and NiMo/Cu-BDC composite. The two sharp characteristic bands at 1386 and 1608 cm^–1^ are indexed to the symmetric and asymmetric stretching modes of −COOH, respectively.^39,40^ The peaks at 829 and 887 cm^–1^ correspond to the out-of-plane and in-plane aromatic C–H bending.^11,41^ The bands at 755 and 1501 cm^–1^ are related to the phenyl ring.^42,43^ The observed peaks at 458 and 567 cm^–1^ can be considered as the stretching vibration peaks of Cu–O.^40,43^ The small peak at about 2900 cm^–1^ is attributed to the aromatic −C–H stretching vibration.^11^ The band at 1666 cm^–1^ is assigned to the carbonyl group in DMF.^44^ However, NiMo/Cu-BDC composite compared to pristine Cu-BDC exhibited a slight blueshift around 500–700 cm^–1^, which suggests that there might be some possible interactions between NiMo and Cu-BDC. Figure S5b illustrates the absorption bands for NiMo NPs before and after heat treatment. The bands located at 650–960 cm^–1^ were ascribed to the vibrations of Mo–O and Mo–O–Mo.^45−47^ Moreover, the FT-IR peaks that appeared at 1287 and 485 cm^–1^ were both due to the Ni–O bonds.^45,48^ After calcination, the FT-IR patterns of Cu-BDC_Cal._ and NiMo/Cu-BDC_Cal._ displayed a vibration peak around 470 cm^–1^ that can be related to Cu–O (Figure S5c).

**Figure 2:**
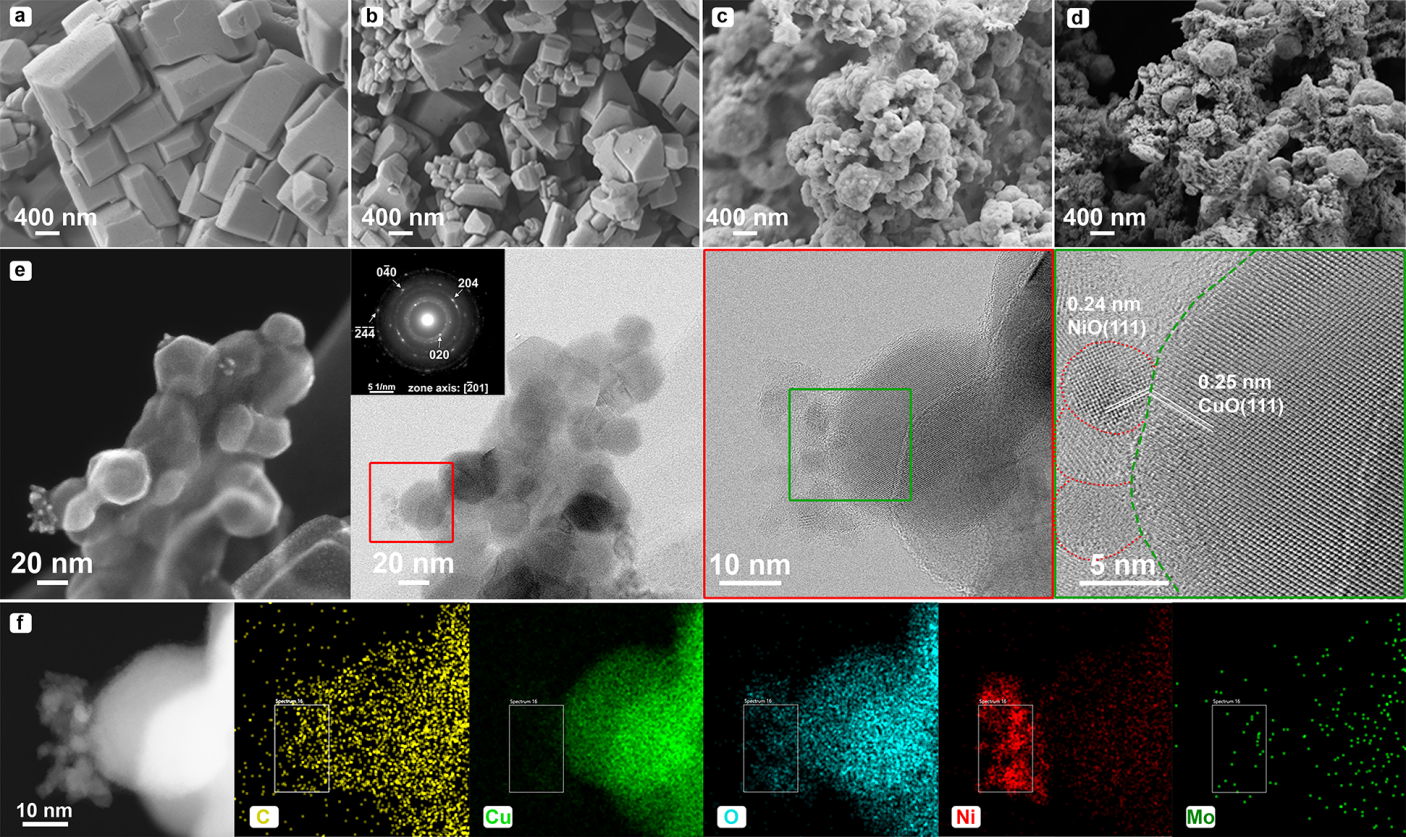
Morphological and microstructural characterizations. SEM images of (a) Cu-BDC, (b) NiMo/Cu-BDC, (c) Cu-BDC_Cal._, (d) NiMo/Cu-BDC_Cal._, (e) STEM, TEM, and HR-TEM images of NiMo/Cu-BDC_Cal._ (inset: SAED pattern), and (f) HAADF-STEM image and the corresponding EDS–STEM mappings of NiMo/Cu-BDC_Cal._.

The morphological characteristics of the specimens were investigated utilizing the FE-SEM technique. The SEM depiction of NiMo before heat treatment is presented in Figure S6, displaying the presence of highly agglomerated nanoscale particles with an approximate dimension of below 20 nm. The SEM micrographs portraying Cu-BDC and NiMo/Cu-BDC, cultivated on the NF backbone, are observable in Figure 2a,b. The micrographs showcase polyhedral structures, their dimensions spanning several hundred nanometers. In contrast, the SEM images of Cu-BDC and NiMo/Cu-BDC post-thermal treatment exhibit noticeable variations relative to their prethermal treatment SEM images. As demonstrated in Figure 2c,d, both samples demonstrate a spherical morphology signified by compact aggregation, attributed to the transition of the Cu-BDC MOF template into CuO@C. Figure S7 presents a better view of the morphology, topography, and uniform distribution of catalysts on the NF. The dimensions of these aggregated particulates are not readily ascertainable. Finally, elemental analysis was performed to observe the composition of the powder samples. As shown in Figure S8, the C content declines significantly following the heat treatment under air, but it does not disappear completely. In addition, the atomic ratio of Ni:Mo in the heat-treated NiMo is 6:1, indicating a significantly low amount of Mo.

Further microstructural features were explored through the utilization of TEM and HR-TEM analyses. The TEM images portraying NiMo NPs arising from post-thermal treatment can be observed in Figure S9a. The assessed size of these nanoparticles is situated below the 10 nm threshold. The HR-TEM images of NiMo following the calcination process disclose exposed crystal planes characterized by a lattice spacing of 0.24 nm (shown in Figure S9b). This feature corresponds to the (111) plane of NiO, aligning favorably with the XRD findings—affirming NiO as the predominant phase. It is important to note that the confirmation of MoO_2_ was not possible due to the limited area covered by the selected batch of samples for TEM.

The TEM micrographs of NiMo/Cu-BDC before the calcination process reveal the presence of thin polygonal layers of Cu-BDC when subjected to the incident electron beam. These smooth layers presented in Figure S10a, forming stacked arrangements, exhibit lateral dimensions in the range of a few hundred nanometers, as corroborated by SEM results. HR-TEM photographs unveil the existence of numerous nanoscale particles situated along the edge of some nanosheet layers, confirming the construction of the nanosheets through the aggregation of these diminutive constituents (see Figure S10b).

Furthermore, the TEM image depicted in Figure S11 pertains to CuO derived from the Cu-BDC MOF, comprising nanosheets and nanoparticles. The measured lattice spacing of 0.25 nm matches with the respective crystallographic plane of (1̅11) within the monoclinic CuO structure. The inset images displayed in Figure S11a and Figure 2e illustrate the SAED patterns of Cu-BDC_Cal._ and NiMo/Cu-BDC_Cal._ along the zone axes [101] and [2̅01], respectively, verifying the formation of a monoclinic CuO structure. Evidencing the interface between NiO nanoparticles (originated from NiMo) and CuO following the calcination process, HR-TEM images within Figure 2e (refer to third and fourth images from left to right) distinctly showcase the interfacial region, characterized by discernible interplanar spacings of 0.24 nm corresponding to the (111) plane of NiO and 0.25 nm associated with the (1̅11) plane of CuO. It is worthy to underscore that HAADF-STEM images of NiMo/Cu-BDC_Cal._ (Figure 2f) confirm the presence of constituent elements including Cu, Ni, Mo, O, and C. Notably, the observed hollow carbonaceous structures in these images are indicative of encapsulating CuO, forming a CuO@C composite configuration.

Metal oxides like TiO_2_, NiO, MoO_2_, and CoO_*x*_, known for their strong water affinity, are commonly utilized in constructing heterostructure catalysts to enhance the water adsorption/dissociation process under alkaline conditions.^49^ For example, Zhang et al.^50^ created a heterostructure catalyst by depositing CoP–CeO_2_ nanosheets on a Ti mesh. The resulting CoP–CeO_2_/Ti catalyst demonstrated enhanced HER performance in alkaline media, achieving a significantly lower overpotential of 43 mV at 10 mA cm^–2^ in 1.0 M KOH compared to CoP/Ti. Density functional theory (DFT) calculations were conducted on the CoP(211) and CoP(211)/CeO_2_(111) systems. It was observed that the interaction at the CoP/CeO_2_ heterointerfaces led to a significant decrease in the energy barrier for water dissociation, reducing it from 1.74 to 1.06 eV.

Furthermore, mixed metal oxides, encompassing two or more oxide components, have been investigated for HER electrocatalysis to harness the advantageous properties resulting from the combination of multiple components.^51^ Liu et al.^52^ synthesized hybrid porous nanosheet arrays comprising tightly interconnected RuO_2_ and NiO NPs. The resulting RuO_2_/NiO composite demonstrated outstanding alkaline HER activity, rivaling that of the benchmark Pt catalyst. The remarkable HER performance was attributed to the potential-induced interfacial synergy between RuO_2_ and NiO: NiO facilitates water dissociation, while RuO_2_-derived Ru actively participates in hydrogen adsorption and evolution.

The presence of a heterojunction at the interface of metal oxide/metal oxide emerges as a pivotal factor in enhancing catalytic activity. Within heterostructured mixed metal oxides (MMO), the heterojunction demonstrates superior HER activity compared to alloys or oxide composites. This superiority is attributed to the heterojunction, which provides MMOs with a more exposed active site than what is observed in alloys or oxide composites.^53^ Wei and colleagues^54^ fabricated concave surface microcubes composed of NiO/Co_3_O_4_ using a MOF precursor (Ni_3_[Co(CN)_6_]_2_). The resulting catalyst facilitated electrolyte penetration, promoting favorable HER kinetics and exhibiting an overpotential of 169.5 mV to achieve 10 mA cm^–2^. Based on the aforementioned theoretical and experimental studies, it is envisaged that the NiO(111)/CuO(1̅11) interface may present a lower energy barrier for the dissociation of HO–H compared to its individual components.

**Figure 3:**
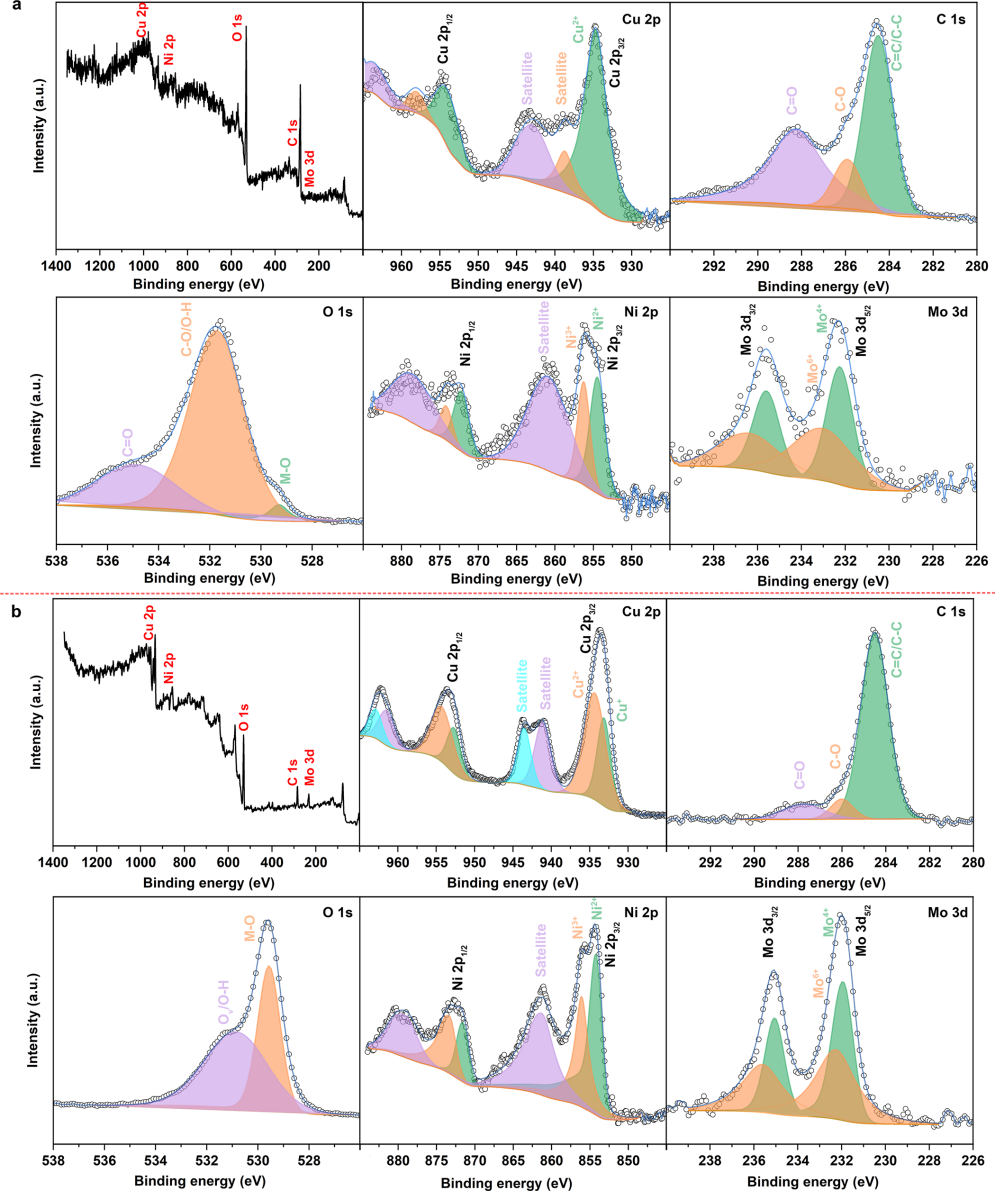
XPS spectra including survey, Cu 2p, C 1s, O 1s, Ni 2p, and Mo 3d for (a) NiMo/Cu-BDC and (b) NiMo/Cu-BDC_Cal._.

To gain a deeper understanding of the surface composition and chemical states of the electrodes fabricated on the NF, XPS analysis was conducted. The XPS survey spectra of both NiMo/Cu-BDC and NiMo/Cu-BDC_Cal._, as depicted in Figure 3, confirmed the presence of elements including Cu, Ni, Mo, C, and O. In Figure 3a, the XPS spectrum of Cu 2p prior to heat treatment revealed a well-fitted representation of the Cu 2p_3/2_ and Cu 2p_1/2_ spin–orbit splitting doublets, featuring distinct peaks at 934.67 and 954.03 eV, which correspond to the Cu^2+^ state.^55^ Subsequent to the calcination process, the Cu 2p spectrum for NiMo/Cu-BDC_Cal._ (Figure 3b) exhibited a transformation into two doublets with spin–orbit values exceeding 19.5 eV. This transformation indicated the coexistence of both Cu^+^ and Cu^2+^ oxidation states, signifying a partial reduction of Cu^2+^ to Cu^+^ following thermal treatment.^5^ This phenomenon was also observed in pristine Cu-BDC (see Figure S12). Notably, the atomic ratios of Cu 2p_3/2_ to Cu 2p_1/2_ for both cases after heat treatment were approximately 0.5.

In the C 1s region of precalcined NiMo/Cu-BDC, three distinct peaks at 284.5 eV (atomic % = 29.26), 285.91 eV (atomic % = 7.69), and 288.24 eV (atomic % = 27.04) eV were observed, corresponding to C=C/C–C, C–O, and C=O bonds, respectively.^56^ Subsequent to heat treatment, the C 1s core-level spectrum displayed three peaks at 284.5 eV (atomic % = 26.25), 286.01 eV (atomic % = 2.34), and 287.78 eV (atomic % = 3.28). The peak that appeared at 284.5 eV is associated with graphitized carbon, which is the dominant carbon species after thermal treatment of the samples. The great attenuation of the peaks corresponding to C–O, and C=O in calcined samples compared to those before heat treatment indicates that most of the oxygen-containing functional groups have been removed.^22,57^ This implies that CuO derived from Cu-BDC MOF may exhibit coordination with the carbon environment. Moreover, for both heat-treated catalysts, C 1s spectra shifted toward higher BEs, that is, the electron is transferred from carbon to Cu and/or Ni/Mo. Hence, the transition metals are partially reduced and endowed with higher electron density, and in turn, a larger number of available active sites for the adsorption of H^+^ increases.^29^ Finally, the O 1s spectra were analyzed, revealing three distinct peaks centered at 529.29, 531.7, and 534.88 eV, which can be attributed to the existence of the Cu–O, C–O/O–H, and C=O.^58,59^ The O 1s XPS spectrum of NiMo/Cu-BDC_Cal._ revealed two peaks located at 530.85 and 531.95 eV, which could correspond to lattice oxygen from CuO and defect centers originating from oxygen deficiency and/or surface-absorbed H_2_O/O–H.^6^ As evident, one of the O 1s XPS peaks disappears after heat treatment, indicating the cleavage of C–O/C=O bonds from the Cu-MOF structure.

Oxygen vacancies can be categorized into two types: surface vacancies and bulk vacancies. Surface vacancies exhibit higher catalytic activity compared to their bulk counterparts, while bulk vacancies demonstrate exceptional stability. Notably, during photocatalytic reactions, surface vacancies contain oxygen species such as O_2_, contributing to their stability.^60^ The literature suggests that the g-factor for oxygen vacancies typically falls within the range of 1.997 to 2.004, a span in line with our measurement results, confirming the existence of oxygen vacancies. (Figure 1d).^60,61^ These g-factors align with expectations, given the limited effects of spin–orbit coupling at the surface. In surface vacancies, the spin contribution remains (S = 1/2), while the orbital contribution is minimal (L = 0). Furthermore, XPS results revealed that, during the reduction of Cu^2+^ to Cu^+^, Ni^3+^ to Ni^2+^, and Mo^6+^ to Mo^4+^, electrons from oxygen vacancies are trapped, leading to the formation of surface oxygen vacancies. Overall, the combined results from EPR spectroscopy and XPS analysis strongly support the existence of oxygen vacancies in both bulk and surface phases across all samples.

The Ni 2p core-level XPS of NiMo/Cu-BDC revealed significant peaks at 854.47, 856.26, and 860.9 eV, corresponding to Ni^2+^, Ni^3+^, and an associated satellite, respectively.^4,16^ Following the calcination process, the Ni 2p spectrum exhibited the same oxidation states of 2+ and 3+ but exhibited a minor shift toward lower BEs, 854.23, 856.08, and 861.31 eV. It can be concluded that the transition metals were subjected to reduction during the heat treatment process. Eventually, Figure 3a,b showcases the Mo 3d region for NiMo/Cu-BDC before and after calcination. Prior to calcination, two sets of doublet pairs were observed at BEs 232.24, 235.59, and 233.03 eV, 236.38 eV. These doublet pairs were attributed to the Mo^4+^ and Mo^6+^ oxidation states, respectively. The sample subjected to heat treatment exhibited the same oxidation states in the Mo 3d spectrum, albeit with a slight shift in BE toward lower values.^62^

### 3.2 Electrocatalytic Studies

**Figure 4:**
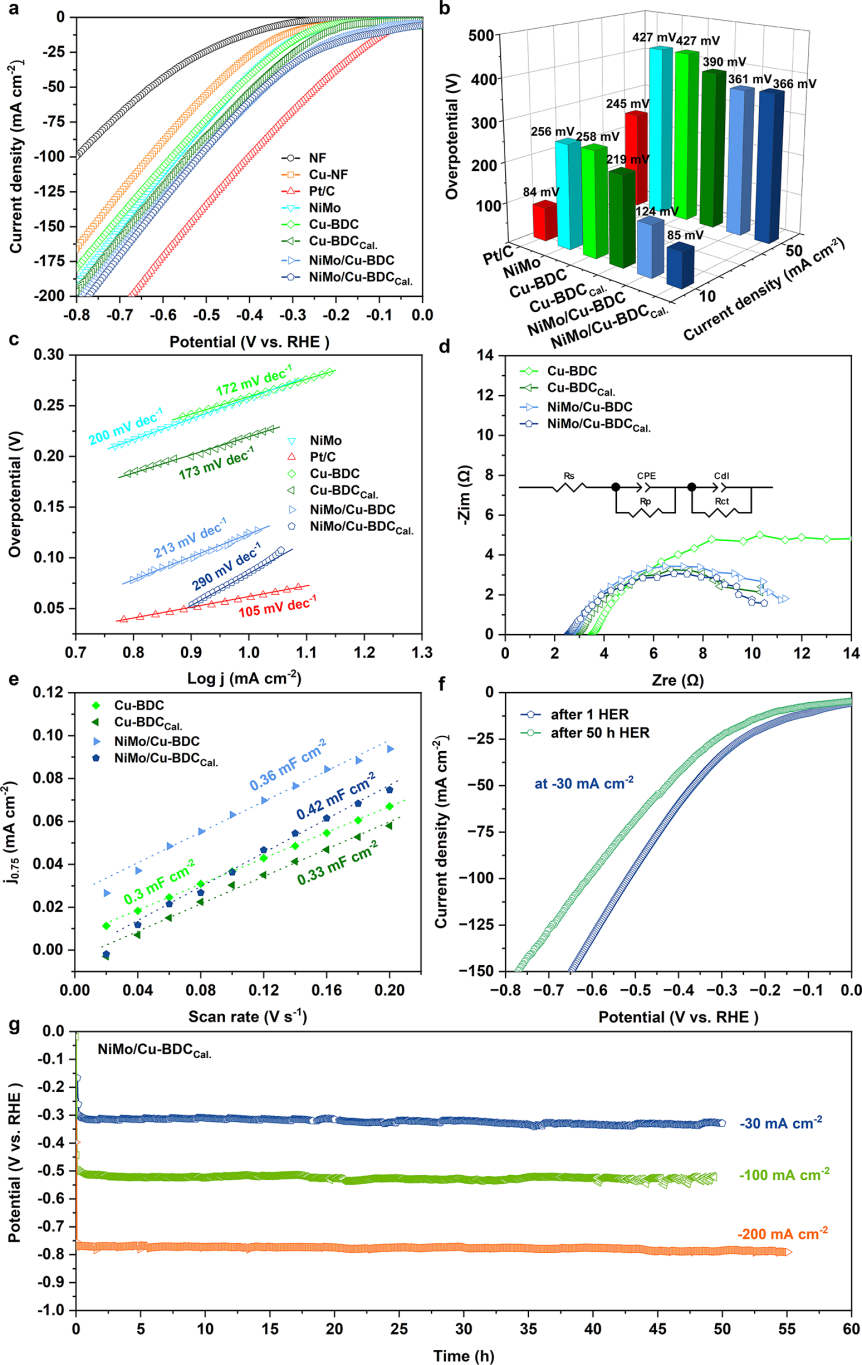
Electrocatalytic hydrogen evolution performances in 1.0 M KOH. (a) HER polarization curves of the samples, (b) 3D bar graphs of overpotentials at 10 and 50 mA cm^–2^, (c) HER Tafel plots obtained by polarization curves, (d) EIS spectra recorded at −300 mV versus RHE, (e) capacitive current density versus scan rate curves for ECSA measurements, (f) HER polarization curves of NiMo/Cu-BDC_Cal._ before and after long-term HER at −30 mA cm^–2^, and (g) long-term HER durability tests of NiMo/Cu-BDC_Cal._ at an applied current densities of −30, −100, and −200 mA cm^–2^.

The prepared electrodes were employed for electrocatalytic HER in 1.0 M KOH aqueous solution at ambient temperature. The LSV curves of fabricated samples along with commercial Pt/C, Cu-coated NF, and NF are shown in Figure 4a. The overpotential at 10 mA cm^–2^ is an index to assess the HER performance of the samples (Figure 4b). As expected, the Pt/C electrode reached 10 mA cm^–2^ at a low overpotential of 83 mV. Among the developed electrodes in this study, the NiMo/Cu-BDC_Cal._ electrode afforded the 10 mA cm^–2^ at an overpotential as low as 85 mV, which is almost identical to that of commercial Pt/C and smaller than that of NiMo/Cu-BDC (126 mV), Cu-BDC_Cal._ (219 mV), NiMo (256 mV), and pristine Cu-BDC (258 mV). Both Cu-BDC_Cal._ (CuO@C) and calcined NiMo (almost NiO phase) did not show very high activity toward alkaline HER. However, their composite product illustrated HER performance similar to Pt/C at low overpotentials. The HER performance of NiMo/Cu-BDC_Cal._ at 10 mA cm^–2^ is comparable to or higher than other reported values for similar catalysts (Table S1). The HER kinetics for the as-prepared samples were measured using corresponding Tafel plots derived from LSVs (Figure 4c). Surprisingly, the best-performing sample exhibited a Tafel slope as high as 290 mV dec^–1^, which is higher than those of Pt/C (105 mV dec^–1^), NiMo/Cu-BDC (213 mV dec^–1^), NiMo (200 mV dec^–1^), Cu-BDC_Cal._ (173 mV dec^–1^), and pristine Cu-BDC (172 mV dec^–1^). All the decisive parameters that can adversely affect the Tafel slope were discussed in our recent publication.^29^ The notable performance of the best-performing HER catalyst can be attributed to (i) high electrical conductivity of Cu-containing species within the sample, (ii) the accelerated electron transfer due to the formation of an interface between the two components, (iii) the electronic redistribution on the catalyst surface as a result of the presence of elements with different electronegativity on the Pauling scale, Cu (1.9), Ni (1.91), Mo (2.16), O (3.44), and C (2.55), and (iv) observation of trapped electrons within the defect structures of NiMo/Cu-BDC_Cal._, enriching the active sites with higher electron density for better proton adsorption.

As reported in previous studies, under alkaline conditions, the kinetics of the HER is elucidated by the adsorption/desorption processes involving hydrogen atoms/molecular hydrogen, employing either Volmer–Heyrovsky (120–40 mV dec^–1^) or Volmer–Tafel (120–30 mV dec^–1^) mechanisms.^63,64^ It is widely acknowledged that the HER kinetics is facile, unaffected by the defined overpotential of the catalyst. Thus, it can be confirmed that the HER kinetics demonstrate a remarkably uniform behavior across all documented catalysts. Therefore, the rate-determining step (RDS) for the as-prepared electrodes is through the Volmer mechanism. Another crucial factor used to evaluate the inherent electrocatalytic performance of materials at the reversible overpotential (η = 0) is referred to as the exchange current density (j^0^), which can be derived by extending the linear segment of the Tafel plot. The exchange current density is a parameter more commonly employed to assess the activity of the HER compared to its use in evaluating the OER. There is a direct correlation between the exchange current density and onset overpotential in the HER.^58,65^ In other words, a catalyst is considered more active when its j^0^ is higher. NiMo/Cu-BDC_Cal._ exhibited a j^0^ value of 5.84 mA cm^–2^, which is twice as NiMo/Cu-BDC (2.64 mA cm^–2^) and much higher compared to Cu-BDC_Cal._ (0.3 mA cm^–2^) and pristine Cu-BDC (0.31 mA cm^–2^).

To further investigate the electrocatalytic performance, the EIS experiment was carried out at −300 mV vs RHE to examine the catalytic kinetics, as portrayed in Figure 4d. The semicircles could be found in the Nyquist plots, which are references to the solution resistance (*R*_s_), porosity resistance (*R*_p_), and charge transfer resistance (*R*_ct_). NiMo/Cu-BDC_Cal._ showed *R*_ct_ around 2.6 Ω, which is smaller than that of its counterparts, namely, NiMo/Cu-BDC (6.2 Ω), and Cu-BDC_Cal._ (4.1 Ω), and pristine Cu-BDC (7.2 Ω). The data resulting from the fitting process is summarized in Table S2. Furthermore, the ECSA of the electrocatalysts was explored by calculating the *C*_dl_ according to the CV results (Figure S13) at different scan rates. From Figure 4e, the results revealed that the NiMo/Cu-BDC_Cal._ possesses higher *C*_dl_ (0.42 mF cm^–2^) than NiMo/Cu-BDC (0.36 mF cm^–2^), Cu-BDC_Cal._ (0.33 mF cm^–2^), and pristine Cu-BDC (0.3 mF cm^–2^), manifesting larger active surface areas and abundant active site numbers for the NiMo/Cu-BDC_Cal._. In this context, ECSA can be obtained through the following equation:^66^
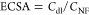
where *C*_NF_ is a *C*_dl_ value of the NF substrate in 1.0 M KOH.^67^ Upon cycling the CV within the same potential window, the *C*_NF_ yielded an impressive electroactive surface area of approximately 0.184 mF cm^–2^. Subsequently, the unitless electroactive surface areas for the various samples were determined, namely, NiMo/Cu-BDC_Cal._ (2.28), NiMo/Cu-BDC (1.95), Cu-BDC_Cal._ (1.79), and pristine Cu-BDC (1.63). Notably, the NiMo/Cu-BDC_Cal._ electrode exhibited the highest ECSA value. To assess the intrinsic activity of the electrodes, the HER performance was normalized based on the respective ECSA values. The ECSA-normalized hydrogen evolution performance is depicted in Figure S14. Remarkably, the best-performing electrode consistently outshines its counterparts, particularly at low overpotentials.

To compare the electroactive surface area with the specific surface area, the BET surface area and pore volume of the selected samples were measured from N_2_ adsorption–desorption isotherms (Figure S15). Both pristine Cu-BDC and NiMo/Cu-BDC demonstrated typical type-I isotherms (Figure S15a,c) according to IUPAC classifications, indicating a microporous nature.^65^ In contrast, the heat-treated catalysts (Figure S15b,d) displayed a type IV isotherm with an H3-type hysteresis loop, suggesting that the pore structures are mainly mesopores.^1^ As shown in Figure S15a,c, the N_2_ adsorption–desorption isotherms for Cu-BDC-containing catalysts are not closed loop. This phenomenon may indicate irreversibility in the adsorption–desorption process. In other words, the adsorbed gas does not completely desorb during the desorption stage, or the desorption process occurs differently than the adsorption process. This phenomenon is often observed in systems with strong interactions between the adsorbent and adsorbate, leading to hysteresis.

The effective BET surface areas (micropore volume) of the pristine Cu-BDC, Cu-BDC_Cal._, NiMo/Cu-BDC, and NiMo/Cu-BDC_Cal._ were measured to be 146.88 m^2^/g (0.061 cm^3^/g), 4.26 m^2^/g (0.001 cm^3^/g), 122.74 m^2^/g (0.049 cm^3^/g), and 14.37 m^2^/g (0.0009 cm^3^/g), respectively. As expected, and explained in the Introduction section, when MOFs are exposed to high temperatures, the degradation or decomposition of the porous structure can be conceived, leading to the framework collapse and dramatic decline in surface area. Additional specifics obtained from the BET analysis were summarized in Table S3. Besides the catalytic activity, the electrocatalytic durability of NiMo/Cu-BDC_Cal._ was evaluated by chronopotentiometry at −30, −100, and −200 mA cm^–2^ in 1.0 M KOH. The catalyst showed no significant decrease in overpotential for 50 h during each long-term exposure to applied currents (Figure 4g), implying its practical long-term durability for the HER. The slight fluctuation of the long-term HER plots is due to the generation of hydrogen bubbles during the reaction.^22,68^ The catalytic activity for the post-HER durability at −30 mA cm^–2^ was assessed through polarization curves, showing that catalytic performance drastically declined from 85 to 170 mV at 10 mA cm^–2^ (Figure 4f).

#### 3.2.1 Post-electrocatalysis Characterization

**Figure 5:**
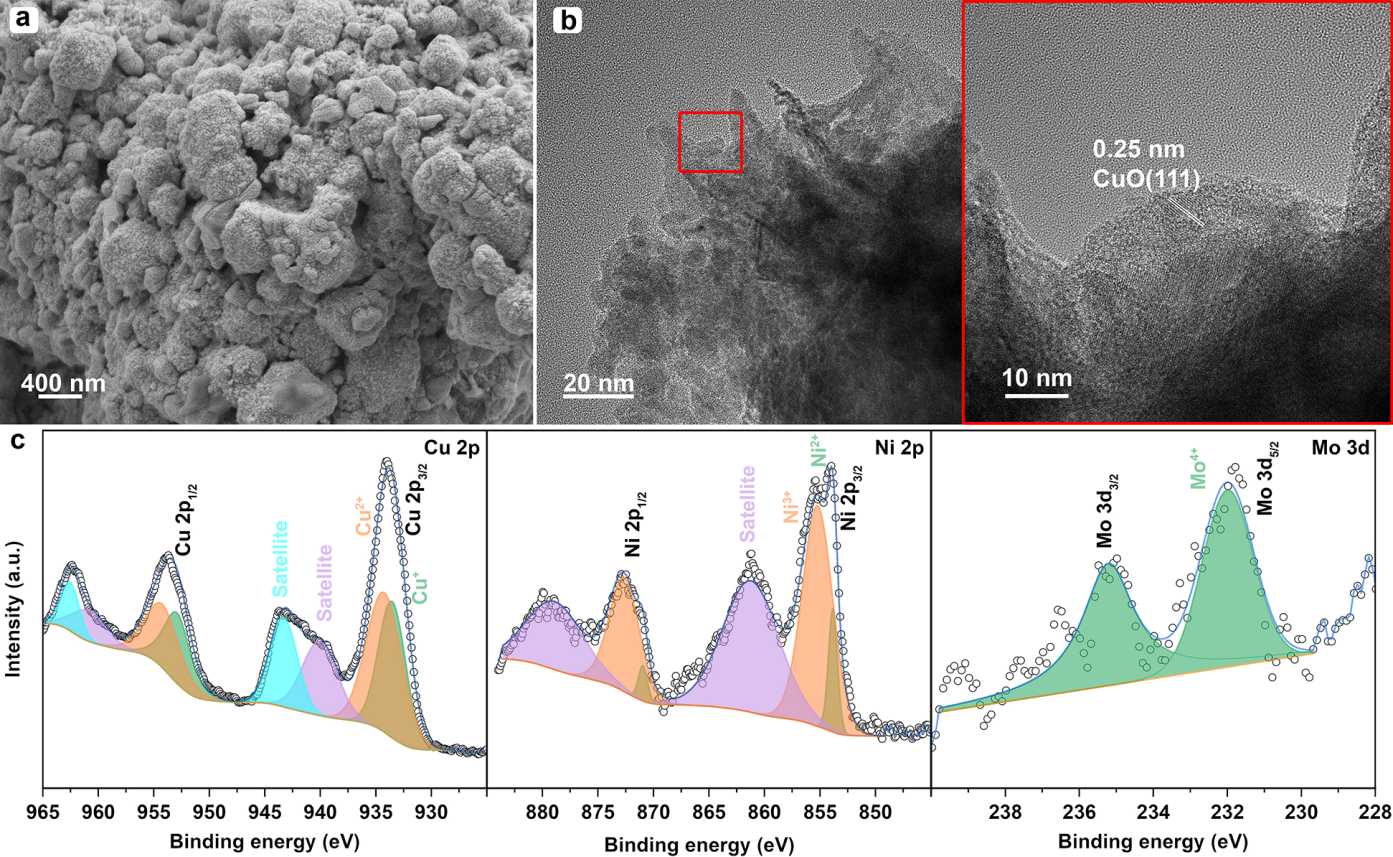
Post-electrolysis characterizations of NiMo/Cu-BDC_Cal._ after long-term HER test at −30 mA cm^–2^. (a) SEM image, (b) TEM and HR-TEM images, and (c) XPS Cu 2p, Ni 2p, and Mo 3d.

To examine the alterations on the electrode after 50 h HER experiment at −30 mA cm^–2^, the post-electrolysis characterizations including SEM, TEM/HR-TEM, and XPS studies were carried out. The SEM images of the HER electrode before and after measurement displayed a distinct difference. The spherical particles tightly stuck to one another, forming big chunks of materials on the NF (refer to the high-magnified SEM image in Figure 5a). Therefore, the porous structure observed before measurement almost disappeared after long-term HER (refer to the low-magnified SEM image in Figure S7d). The TEM/HR-TEM micrographs demonstrated similar morphology for the used sample after a long-term durability test, showcasing nanoparticles and nanosheets within the sample batch. From Figure 5b, the dense agglomeration of nanoparticles after stability measurement is discernible with the darker region in the TEM images surrounded by a comparatively lighter region. XPS results showed that the transition metals including Cu, Ni, and Mo moved to the lower binding energies compared to the initial sample. Interestingly, for the Cu 2p XPS core spectrum, the Cu^+^ shifted to higher BEs, while Cu^2+^ shifted to lower BEs. In addition, the valence states of Mo 3d changed, transforming into only Mo^4+^ oxidation state, eliminating Mo^6+^. It can be concluded that during long-term HER, Ni 2p, and Cu 2p were electrochemically stable. On the contrary, Mo 3d was not completely stable during the 50 h HER operation (see Figure 5c). The comparison of atomic proportions for the best-performing HER electrode before and after the stability test revealed that C 1s and O 1s remained almost unchanged. In sharp contrast, transition metals such as Cu 2p (12. 27% → 7.83%), Ni 2p (5.71% → 1.8%), and Mo 3d (1.78% → 0.11%) dramatically declined after the 50 h HER test. These findings suggested that transition metals in the framework of the catalyst were the favorable active sites for the adsorption of H^+^ and its subsequent evolution to H_2_.

Further investigating the electrode’s stability, an analysis was conducted following a 50 h HER scan at −100 mA cm^–2^. XRD analysis, performed on the bulk form of the electrode, revealed prominent reflections associated with Ni and Cu (refer to Figure S16a). To delve into surface degradations, XPS analysis was employed. Surprisingly, the findings differed from those observed during the long-term HER at −30 mA cm^–2^. The Cu 2p XPS spectrum exhibited metallic Cu at 932.1 eV (2.32%) and Cu^+^ at around 933.2 eV (1.7%). Additionally, Ni 2p XPS showed only the oxidation state of Ni^2+^ at 854.9 eV (0.96%), possibly originating from Ni(OH)_2_. Finally, the Mo 3d XPS core-level spectrum displayed a solitary oxidation state of Mo^4+^, accompanied by a notably noisy signal, indicating its insignificant content on the surface of the electrode (see Figure S16b).

### 3.3 Proposed Mechanism

The superior HER performance of NiMo/Cu-BDC_Cal._ can be attributed to the synergy between the high charge mobility of the carbon layer formed at high temperatures, the charge flow between NiO—originated from NiMo—and CuO via the *in situ* formed interface, and the catalytically active metal sites enriched with high electron clouds in the proximity of ionized oxygen vacancies.

The coexistence of metallic Cu (electrodeposited on the NF) and carbon layer establishes a conductive network for the rapid charge flow in the redox reaction. Moreover, the carbon layer could effectively reduce the dense aggregation of metal oxides during high-temperature processes, creating a porous structure for better contact with water and detachment of bubbles.^6,22^ The close contact between the carbon layer and NiO/CuO facilitates the electron transfer to the catalytically active metal sites.

Based upon XPS results, both NiO and CuO possess partially negative charges after the coupling of two components (refer to XPS interpretation, Figure 3). Hence, the charges flow from the carbon layer to NiO and CuO, resulting in higher electron density on Ni and Cu. NiO_1–*x*_ has been shown to play a vital role in the HER process.^69−71^ It is proved that transition metal oxides are not suitable catalysts for transforming the H^+^ to H_2_.^72^ Yet, it is proposed that the dissociation of water (H^+^/OH^–^) is more favorable on metal oxides (more favorable Volmer kinetics), on the other hand, H_2_ is more readily formed on a metallic surface.^29^ As outlined in prior experimental and theoretical studies, the catalytic rate of the HER in an alkaline solution, governed by both the activation energy barrier for water dissociation and the adsorption energy of hydrogen intermediates (H*), is approximately 2 orders of magnitude lower in an alkaline environment compared to that in an acidic electrolyte (e.g., on a Pt surface).^73,74^

As mentioned earlier, upon the removal of Mo from the amorphous structure of NiMo, Mo reacts with and binds to oxygen. Consequently, the resulting NiO exhibits oxygen deficiency, leading to the designation of the final product as NiO_1–*x*_. This oxygen vacancy modification introduces new electronic states near the Fermi level, directly enhancing the electronic conductivity of NiO. The introduction of oxygen vacancy in NiO_1–*x*_ has been demonstrated to facilitate facile water dissociation, aligning with the existing literature.^38^ As a result, the engineered NiO/CuO proves to be an effective electrocatalyst: NiO aids in water dissociation, and CuO-derived Cu actively participates in the adsorption and desorption of hydrogen.

**Scheme 2:**
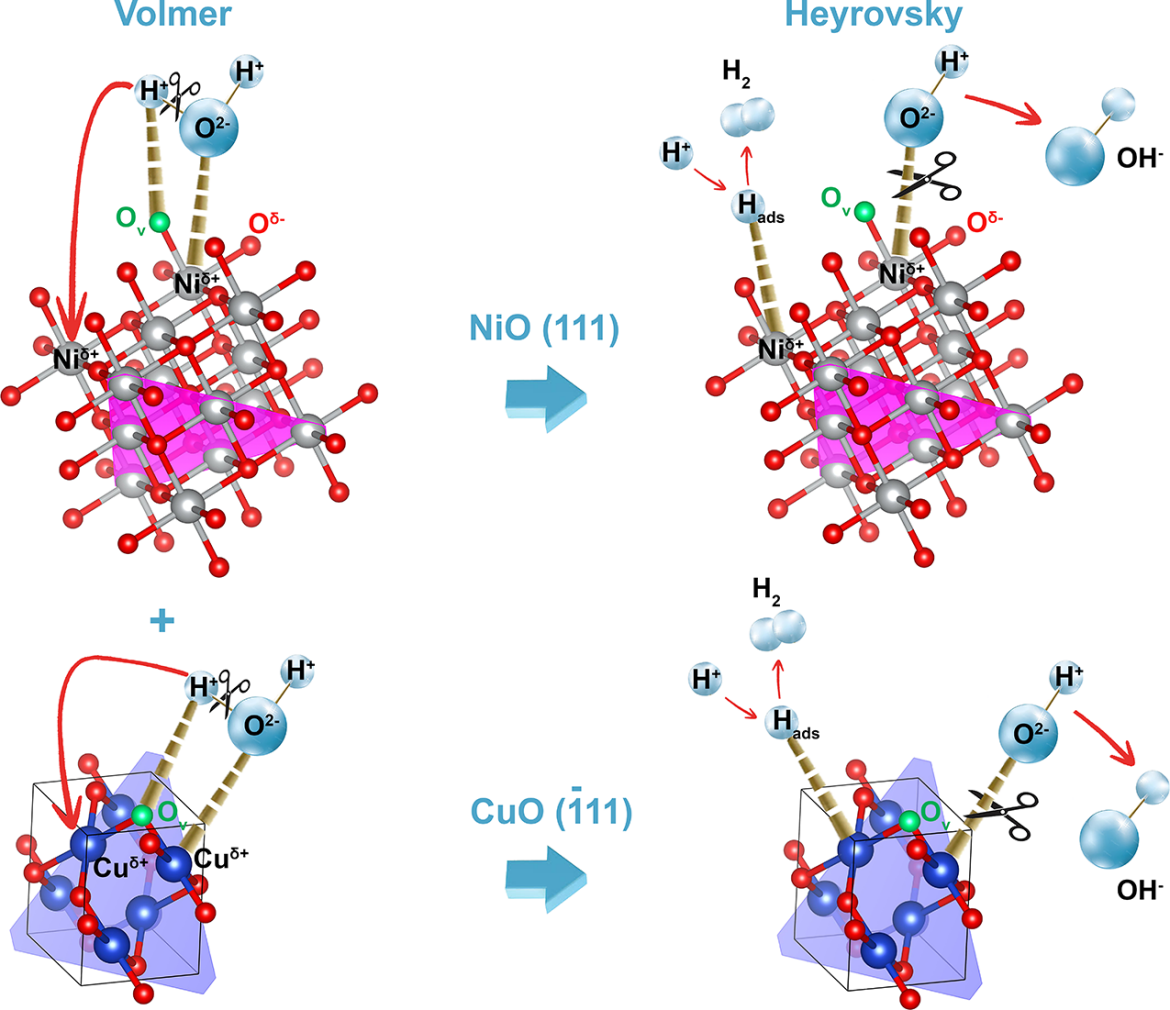
Schematic Presentation of the Proposed HER Mechanism

Considering the Tafel slopes, which are above 120 mV dec^–1^, the HER is primarily governed by a Volmer step, followed by a Heyrovsky step. As illustrated in Scheme 2, in the first step, interactions—that are electrostatic attraction between Cu^δ+^/Ni^δ+^ on the catalyst surface with O^2–^ ions, along with the interaction between O_v_ on the catalyst surface with H^+^ ions, facilitate the adsorption of H_2_O and weaken the H–O–H bond, leading to the dissociation of adsorbed H_2_O into OH^–^ and H^+^ ions. In the second step, the generated OH^–^ ions, resulting from the dissociation of water, coordinate with Cu^δ+^/Ni^δ+^ ions in the vicinity of oxygen (O), while H^+^ ions adsorbed (ads) on O_v_ ions may undergo surface diffusion—that is, they can move along the surface of the crystal lattice, hopping from one adsorption site to another and transfer to nearby Cu^δ+^/Ni^δ+^ ions. The adsorption of another water molecule and the combination of two H^+^ ions result in the formation of molecular H_2_.

## 4 Conclusions

In brief, a nanocomposite electrocatalyst, NiO/CuO@C derived from NiMo/Cu-BDC MOF, was fabricated on Cu-coated NF through hydrothermal procedure followed by calcination under air. The TEM and HR-TEM micrographs revealed the formation of a tight nanostructured interface between NiO (originated from NiMo) and CuO (derived from Cu-BDC). This can expose the active metallic sites, alleviate the adsorption of intermediates (H^+^/OH^–^), and boost the diffusion of ions. Moreover, the carbon layer could improve the charge transfer through the structure. Thus, the metals were endowed with higher electron density to adsorb the H^+^. Furthermore, the higher conductivity of the product led to lower charge transfer resistance on the electrode/electrolyte interface. The fabricated electrode afforded 10 mA cm^–2^ at a small overpotential of 85 mV toward hydrogen evolution in 1.0 M KOH solution. Finally, the chronopotentiometry experiment was carried out at −30, −100, and −200 mA cm^–2^ for 50 h each to evaluate the catalyst’s long-term durability. The fabricated electrode demonstrated remarkable durability at high current densities. The EPR and XPS results proved the formation of oxygen vacancy both on the surface and in the bulk, indicating the high electron density around oxygen deficiency that could accelerate the adsorption of H^+^ during the Volmer step. While the oxygen vacancy-modified NiO alleviates the cleavage of water, CuO-derived Cu improves the evolution of hydrogen during the Heyrovsky step. This work can be used to develop cheap metal oxide nanocomposites that not only are active but also stable under high current densities.

## Supporting Information Available

Comprehensive information about the experimental procedure, the optical microscope image, XRD patterns, crystal structures of Cu-BDC, Raman spectra, FT-IR spectra, the SEM/EDS images, TEM/HR-TEM micrographs, XPS, ECSA, ECSA-normalized HER, electrochemical performance tables, and BET adsorption–desorption isotherms (PDF)
