# Supplementary material for: In Situ Design of a Nanostructured Interface between NiMo and CuO Derived from Metal–Organic Framework for Enhanced Hydrogen Evolution in Alkaline Solutions
Source: ACS Appl Mater Interfaces. 2024 Feb 20;16(8):10078–92. doi: 10.1021/acsami.3c17588 (PMC10910462; doi:10.1021/acsami.3c17588)
Supplement: Supplementary file 1 — am3c17588_si_001.pdf [file am3c17588_si_001.pdf]

Supporting Information

In Situ Design of a Nanostructured Interface between NiMo and CuO Derived from Metal-Organic Framework for Enhanced Hydrogen Evolution in Alkaline Solutions

Ebrahim Sadeghi[1,2], Sanaz Chamani[1], Ipek Deniz Yildirim[3], Emre Erdem[3,4], Naeimeh Sadat Peighambardoust[1], and Umut Aydemir[1,5,*]

[1] Koç University Boron and Advanced Materials Applications and Research Center (KUBAM), Sariyer, Istanbul, 34450, Turkey

[2] Graduate School of Sciences and Engineering, Koç University, Sariyer, Istanbul, 34450, Turkey

[3] Faculty of Engineering and Natural Sciences, Materials Science and Nano Engineering, Sabanci University, Istanbul 34956, Turkey

[4] Sabanci University Integrated Manufacturing Technologies Research and Application Center, Composite Technologies Center of Excellence, Teknopark Istanbul, Pendik, Istanbul 34906, Turkey

[5] Department of Chemistry, Koç University, Sariyer, Istanbul, 34450, Turkey

* Corresponding author; Email: uaydemir@ku.edu.tr

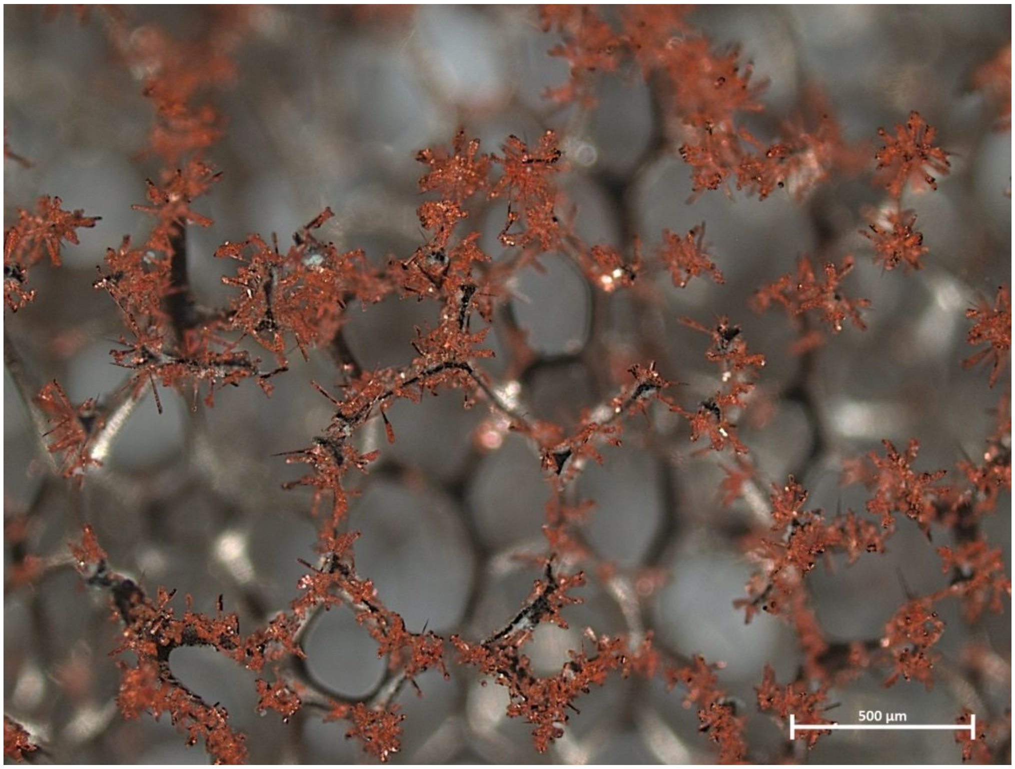

Figure S1. Polarized optical microscope image of Cu-coated NF using electrodeposition method.

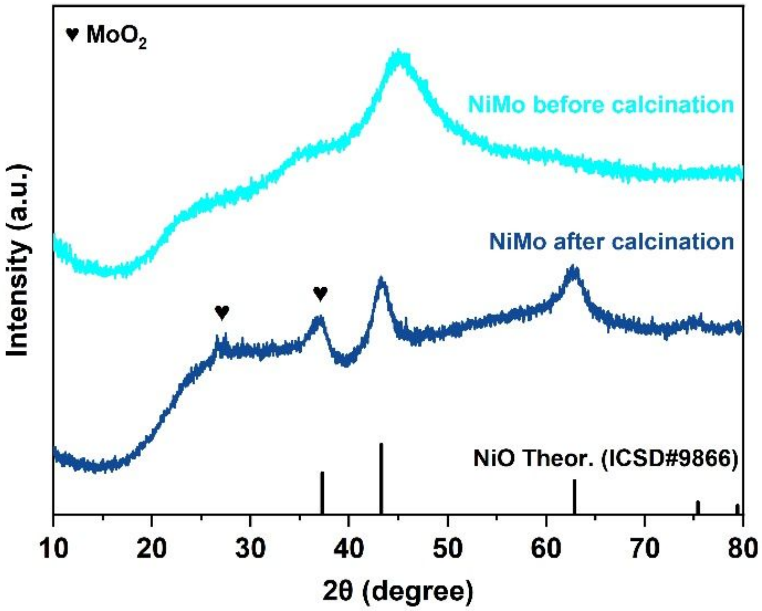

Figure S2. XRD patterns of NiMo nanoparticles before and after the calcination process at 500 °C under air.

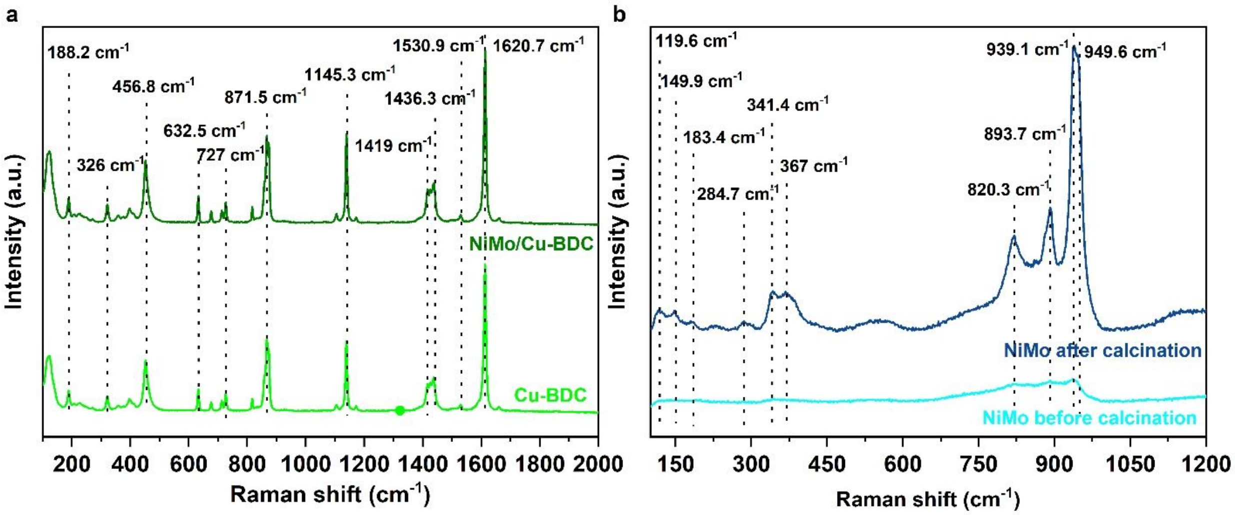

Figure S3. Raman spectra for a) Cu-BDC and NiMo/Cu-BDC and b) NiMo nanoparticles before and after calcination process at 500 °C under air.

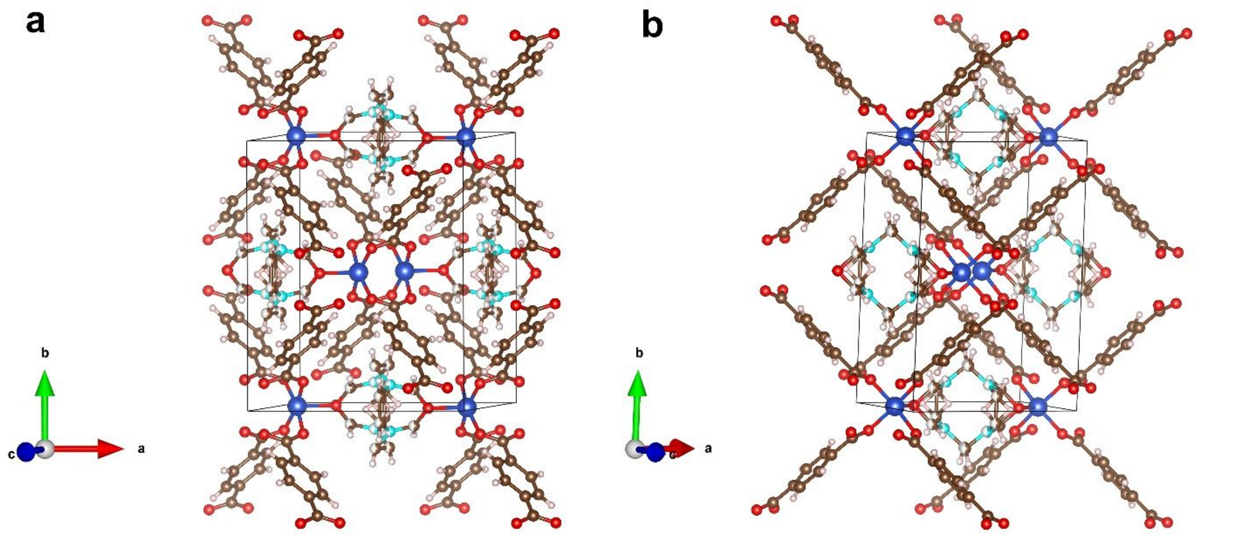

Figure S4. a and b) Crystal structures of Cu-BDC MOF from two perspectives, Cu (dark blue), C (dark brown), H (pink), O (red), and N (light blue).

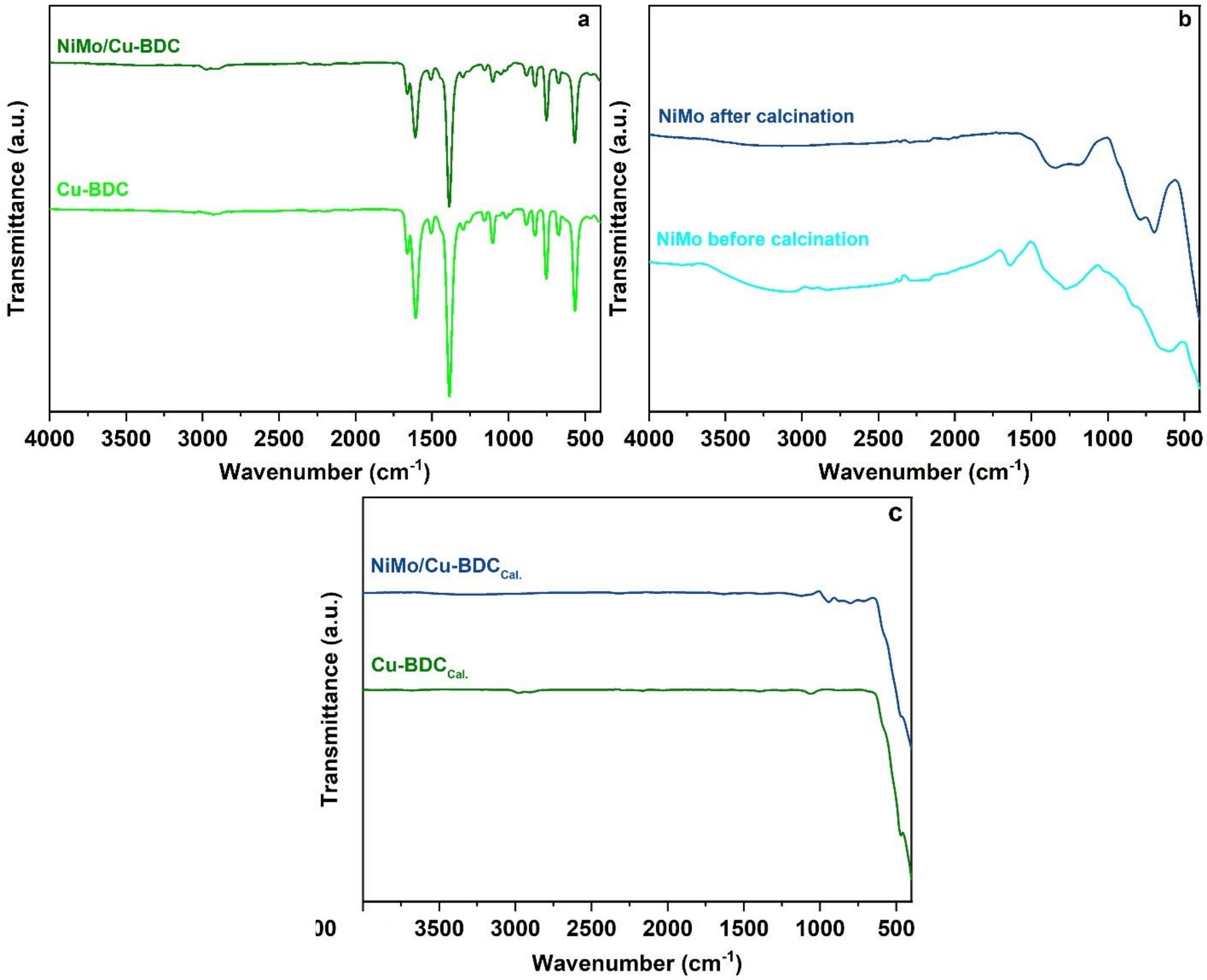

Figure S5. Ft-IR spectra for a) Cu-BDC and NiMo/Cu-BDC and b) NiMo nanoparticles before and after calcination process, and c) Cu-$BDC_{Cal.}$ and NiMo/Cu-$BDC_{Cal.}$.

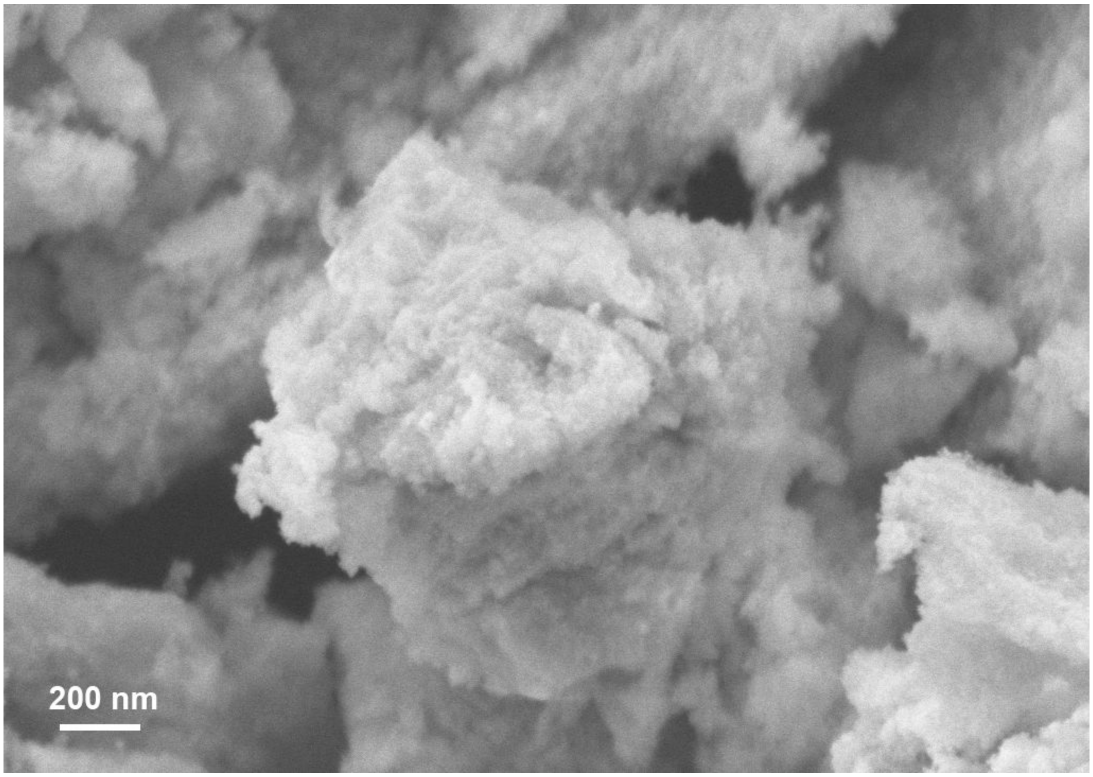

Figure S6. SEM image of NiMo nanoparticles before calcination process.

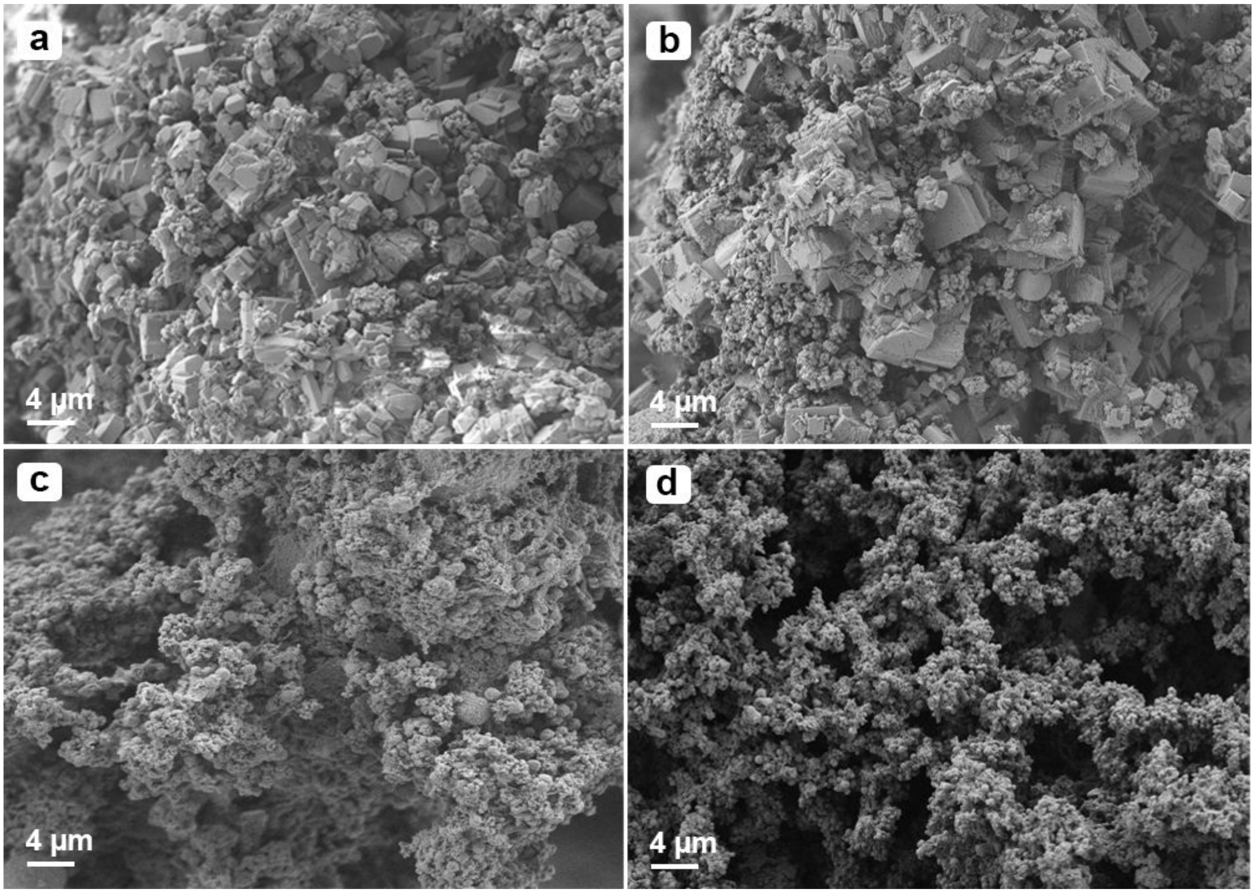

Figure S7. Illustration of uniform growth of catalysts on the backbone of NF substrate. a) Cu-BDC, b) Cu-$BDC_{Cal.}$, c) NiMo/Cu-BDC, and d) NiMo/Cu-$BDC_{Cal.}$.

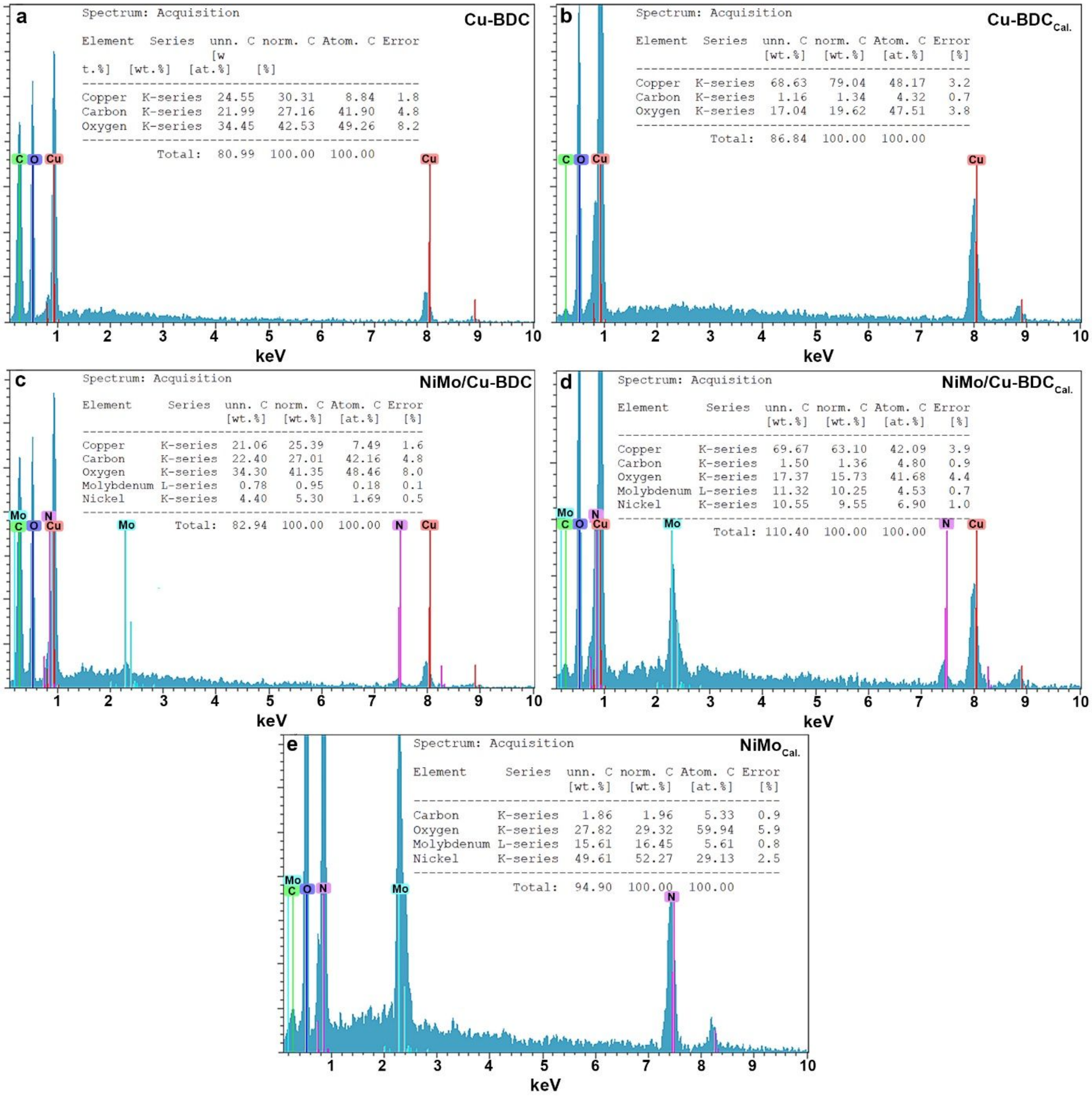

Figure S8. Energy dispersive x-ray spectroscopy analysis for the as-prepared samples.

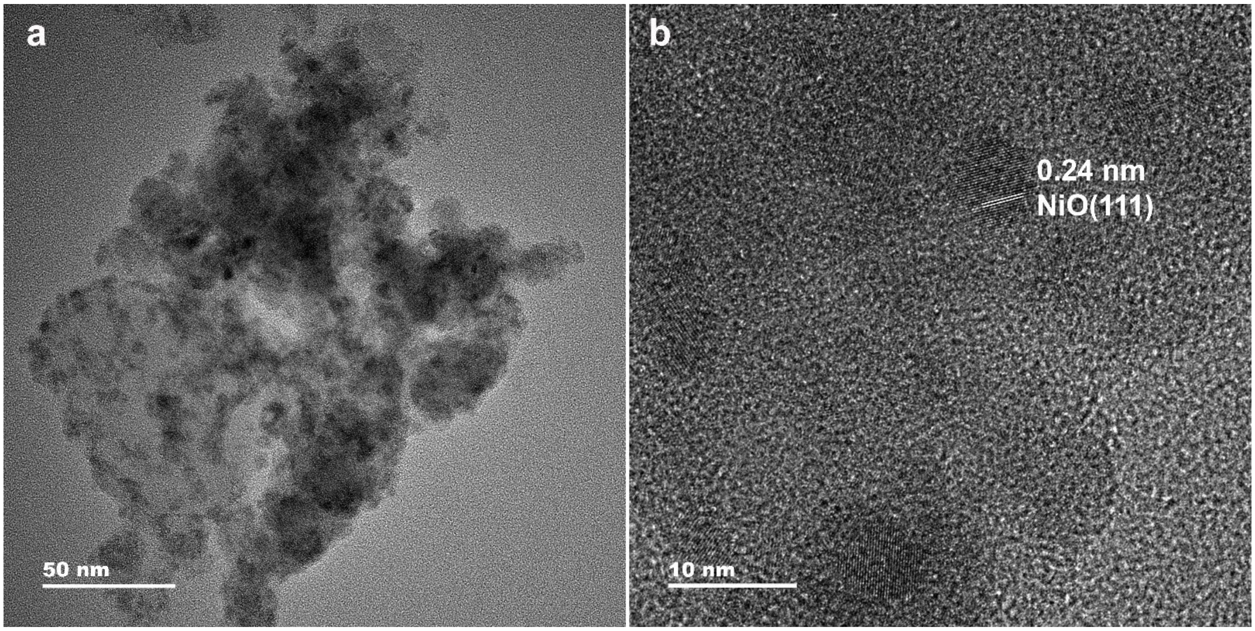

Figure S9. a) TEM and b) HR-TEM images of NiMo after calcination process at 500 °C under air.

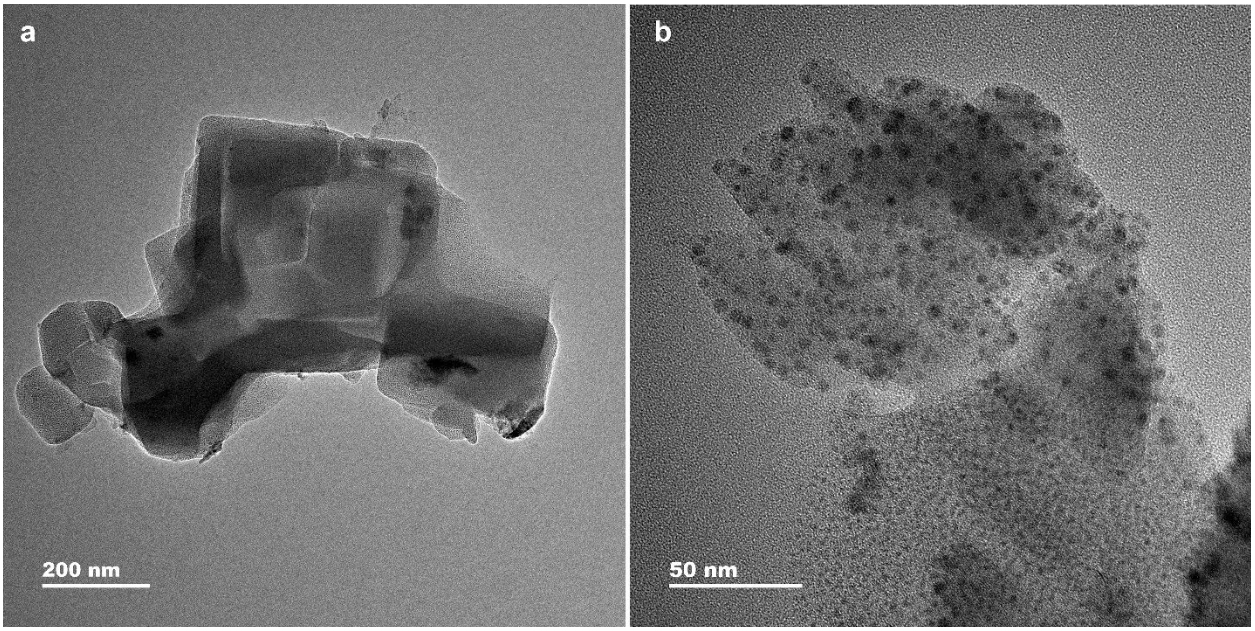

Figure S10. a) TEM and b) HR-TEM images of NiMo/Cu-BDC.

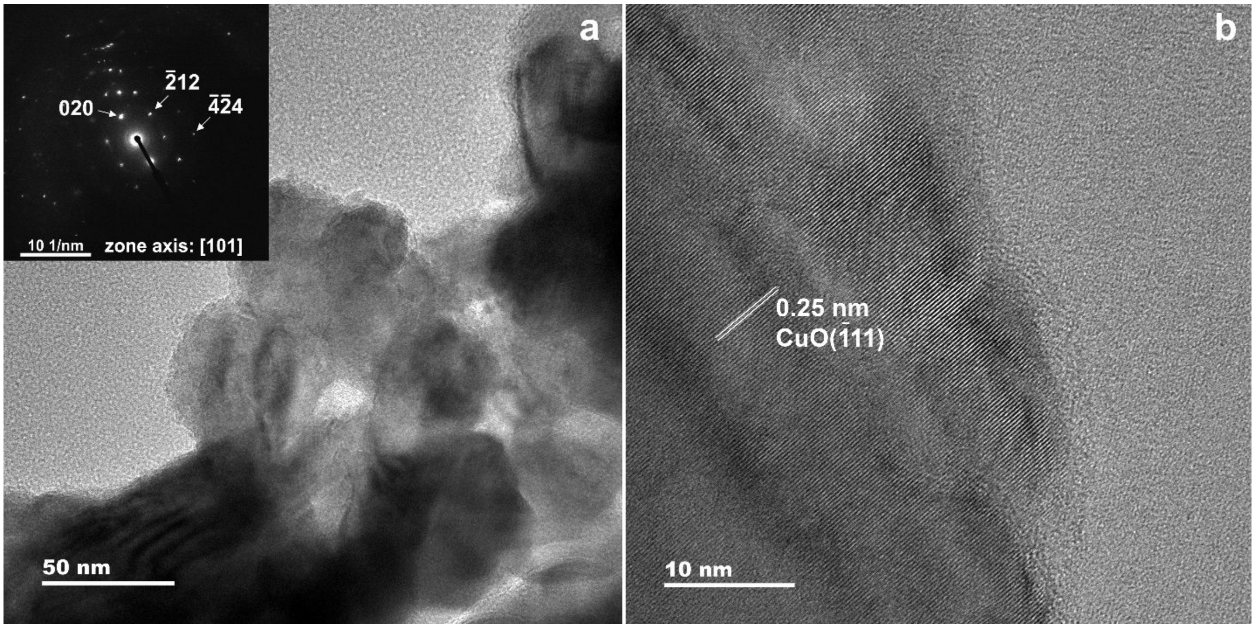

Figure S11. a) TEM and b) HR-TEM images of Cu-BDC$_{Cal.}$ (inset: SAED image).

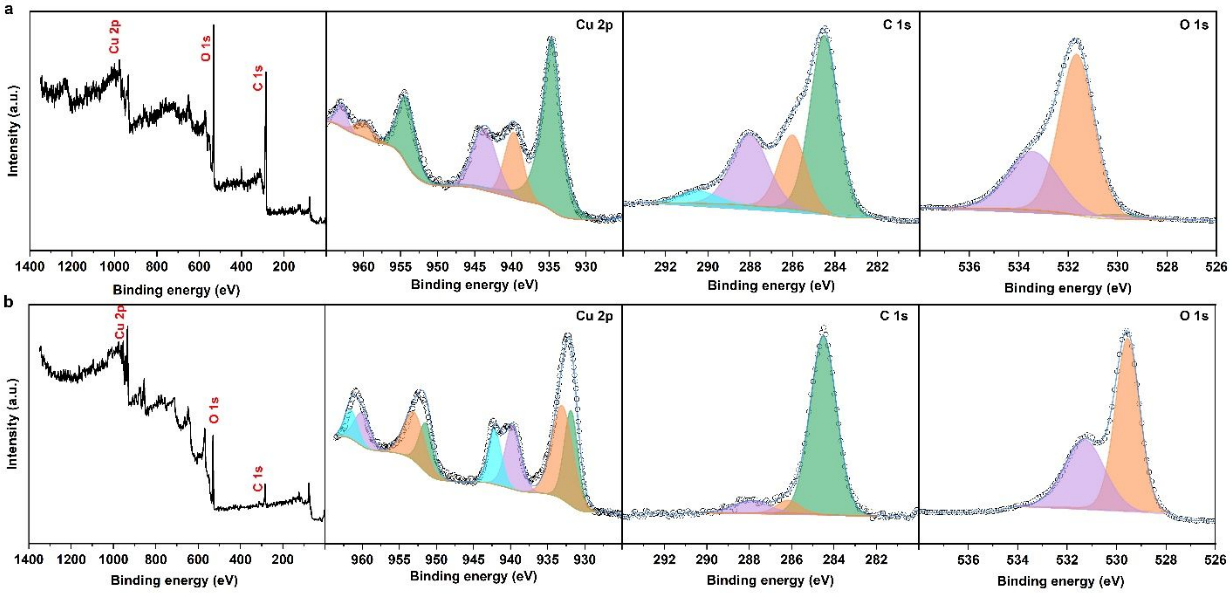

Figure S12. XPS spectra including survey, Cu 2p, C 1s, and O 1s for a) Cu-BDC and b) Cu-BDC$_{Cal.}$.

Table S1. The HER performance of recent reports on similar electrocatalysts at 10 mA cm^{-2}.

**Electrocatalyst**	**Electrolyte**	**η^{HER} (mV) @ 10 mA**	**Ref.**
Co_3O_4@CuO	KOH	288	[1]
CuO/ Cu_2O@C	KOH	121	[2]
Cu/CuO@Co/Co_3O_4	KOH	81	[3]
CuS@C<br>Cu@C	KOH	128<br>370	[4]
NiO/MoO_2	KOH	48	[5]
C–NiCu-BDC-GO	KOH	400	[6]
CuCo-BET@rGO	KOH	120	[7]
Co-Ni-Se/C	KOH	89	[8]
NiO/Co_3O_4	KOH	169.5	[9]
NiMo/CuO@C derived from Cu-MOF	KOH	85	This work

Table S2. Fit parameters from the equivalent circuit for as-fabricated electrodes.

Sample	R_s (Ω)	R_p (Ω)	CPE-T (F^{-1} s^{1-n})	R_{ct} (Ω)	C_{dl}-T (F^{-1} s^{1-n})
**Cu-BDC**	3.508	12.69	0.21038	7.242	0.068377
**Cu-BDC$_{Cal.}$**	2.767	6.653	0.030981	4.146	0.48013
**NiMo/Cu-BDC**	2.6	7.19	0.030728	6.211	0.54241
**NiMo/Cu-BDC$_{Cal.}$**	2.524	6.282	0.032484	2.608	0.21292

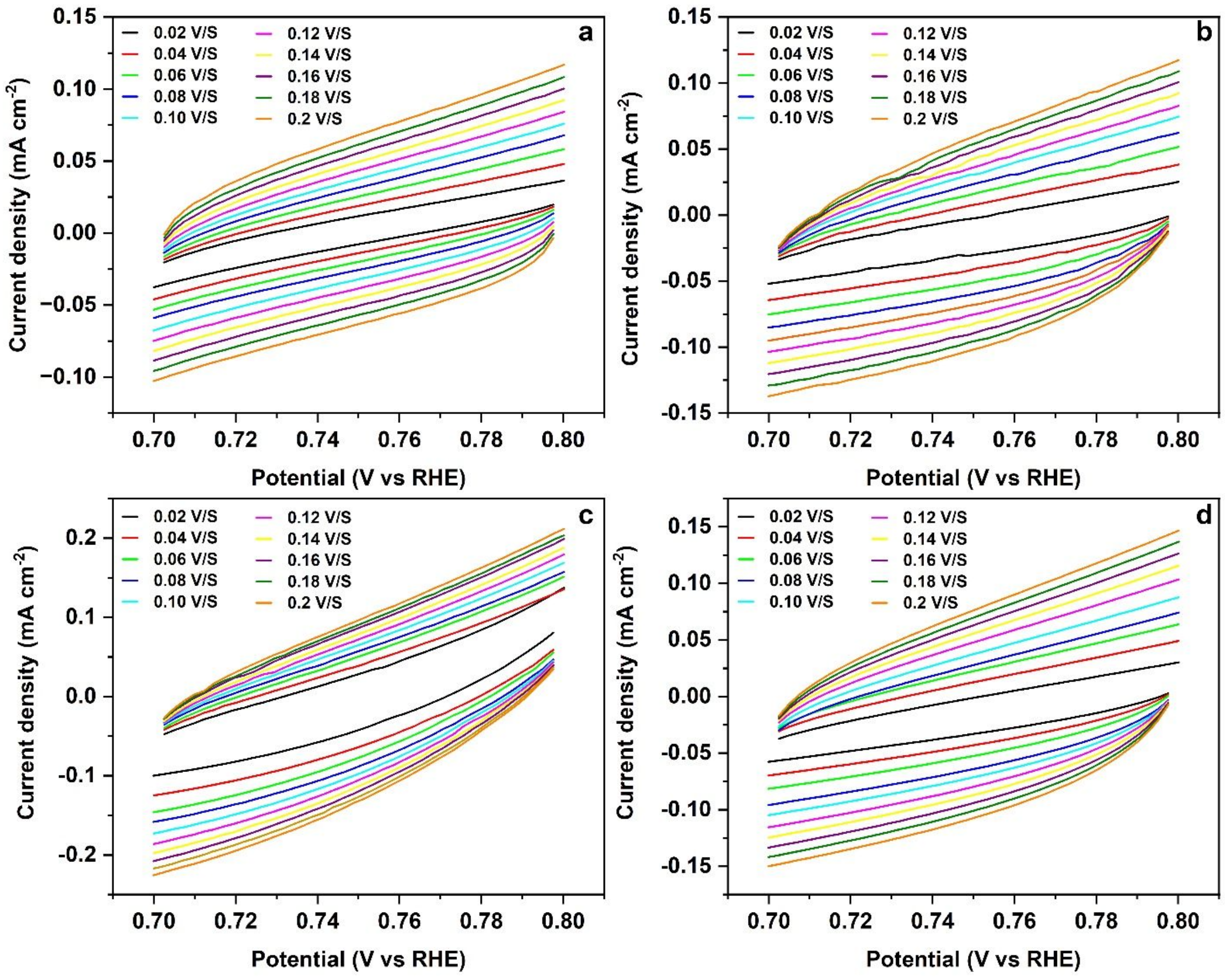

Figure S13. Typical cyclic voltammetry (CV) curves obtained at different scan rates (0.02–0.2 V s^{-1}) within the potential window 0.7–0.8 V vs RHE for a) Cu-BDC, b) Cu-BDC$_{Cal.}$, c) NiMo/Cu-BDC, and d) NiMo/Cu-BDC$_{Cal.}$.

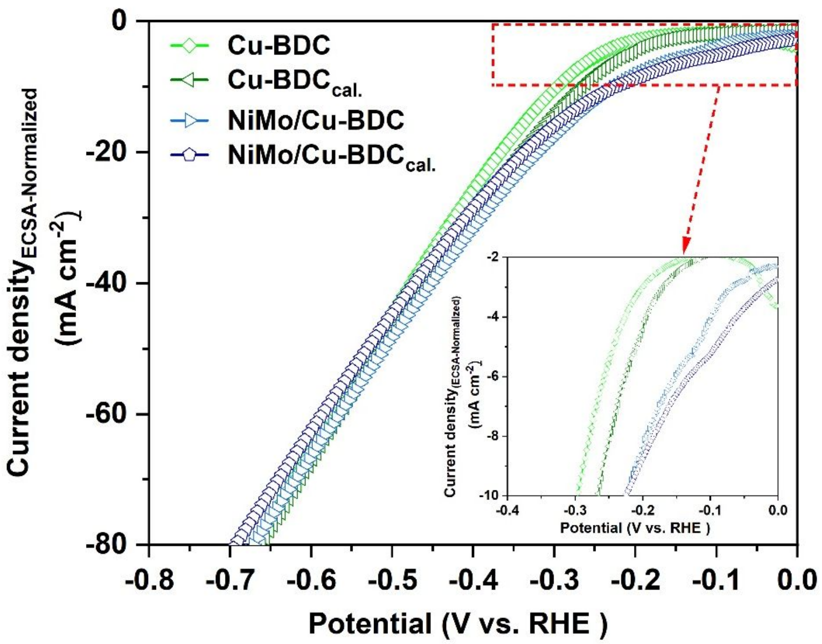

Figure S14. HER polarization curves of the samples normalized to the ECSA.

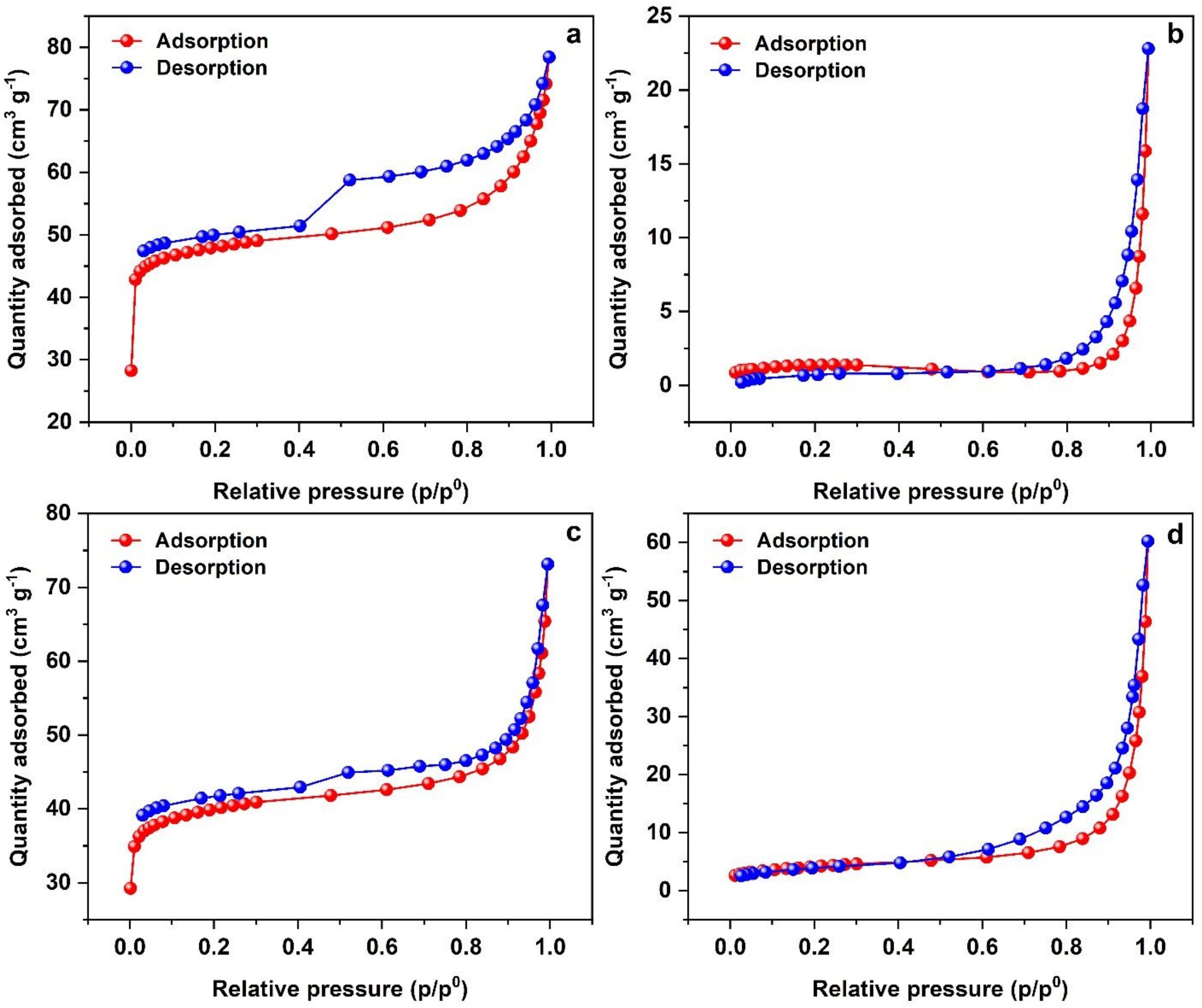

Figure S15. N_2 adsorption-desorption isotherms of a) Cu-BDC, b) Cu-BDC$_{Cal.}$, c) NiMo/Cu-BDC, and d) NiMo/Cu-BDC$_{Cal.}$.

Table S3. Further details derived from BET analysis of the samples.

**Sample**	**BET Surface Area (m^2/g)**	**Micropore Area (m^2/g)**	**External Surface Area (m^2/g)**	**Micropore Volume (cm^3/g)**
**Cu-BDC**	146.88	117.99	28.88	0.061
**Cu-BDC$_{Cal.}$**	4.26	2.49	1.77	0.001
**NiMo/Cu-BDC**	122.74	94.59	28.15	0.049
**NiMo/Cu-BDC$_{Cal.}$**	14.37	1.99	12.37	0.0009

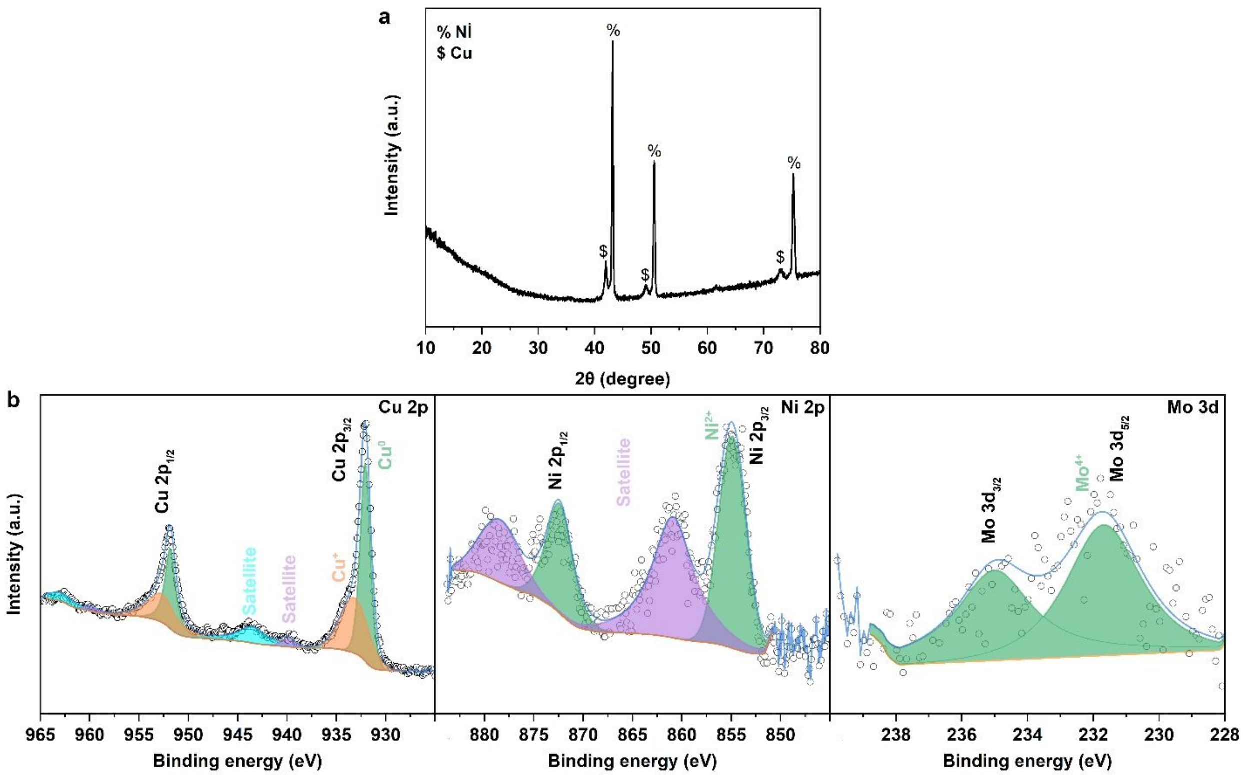

Figure S16. Post-electrolysis characterizations of NiMo/Cu-BDC$_{Cal.}$ after long-term HER test at -100 mA cm^{-2}. a) XRD pattern from the bulk electrode and b) XPS Cu 2p, Ni 2p, and Mo 3d.

References

1. Tahira, A.; Ibupoto, Z. H.; Willander, M.; Nur, O., Advanced Co3o4ecuo Nano-Composite Based Electrocatalyst for Efficient Hydrogen Evolution Reaction in Alkaline Media. *Int J Hydrogen Energy* **2019,** *44* (48), 2614.
2. Zhang, L.; Wang, C.; Jiu, H.; Xu, Q.; Li, X.; Song, W.; Luo, S.; Zhao, J., Metal–Organic Framework Derived Carbon-Encapsulated Hollow Cuo/Cu 2 O Heterostructure Heterohedron as an Efficient Electrocatalyst for Hydrogen Evolution Reaction. *Dalton Transactions* **2022,** *51* (8), 3349-3356.
3. Cai, Z.; Li, A.; Zhang, W.; Zhang, Y.; Cui, L.; Liu, J., Hierarchical Cu@ Co-Decorated Cuo@ Co3o4 Nanostructure on Cu Foam as Efficient Self-Supported Catalyst for Hydrogen Evolution Reaction. *Journal of Alloys and Compounds* **2021,** *882*, 160749.
4. Rong, J.; Xu, J.; Qiu, F.; Fang, Y.; Zhang, T.; Zhu, Y., 2d Metal-Organic Frameworks-Derived Preparation of Layered Cus@ C as an Efficient and Stable Electrocatalyst for Hydrogen Evolution Reaction. *Electrochimica Acta* **2019,** *323*, 134856.
5. Zhou, Y.-N.; Li, M.-X.; Shi, Z.-N.; Zhou, J.-C.; Dong, B.; Jiang, W.; Liu, B.; Yu, J.-F.; Chai, Y.-M., Crystal–Amorphous Nio/Moo 2 with a High-Density Interface for Hydrogen Evolution. *Inorganic Chemistry Frontiers* **2022,** *9* (9), 2087-2096.
6. Jia, L.; Wagner, P.; Chen, J., Electrocatalyst Derived from Nicu–Mof Arrays on Graphene Oxide Modified Carbon Cloth for Water Splitting. *Inorganics* **2022,** *10* (4), 53.
7. Song, Y.; He, H.; Zhao, Y.; Li, Y.; Wu, M.; Li, J.; Lu, X.; Zhao, L.; Wei, L., Effective Construction of a Cuco Mof@ Graphene Functional Electrocatalyst for Hydrogen Evolution Reaction. *Dalton Transactions* **2023,** *52* (36), 12695-12703.
8. Ming, F.; Liang, H.; Shi, H.; Xu, X.; Mei, G.; Wang, Z., Mof-Derived Co-Doped Nickel Selenide/C Electrocatalysts Supported on Ni Foam for Overall Water Splitting. *Journal of Materials Chemistry A* **2016,** *4* (39), 15148-15155.
9. Wei, X.; Zhang, Y.; He, H.; Gao, D.; Hu, J.; Peng, H.; Peng, L.; Xiao, S.; Xiao, P., Carbon-Incorporated Nio/Co 3 O 4 Concave Surface Microcubes Derived from a Mof Precursor for Overall Water Splitting. *Chemical Communications* **2019,** *55* (46), 6515-6518.
